# Bridging Energy Storage and Electrochemical Sensing: A Bibliometric and Critical Analysis of Metal, Metal Oxide, and Carbon-Based Nanocomposites

**DOI:** 10.3390/nano16191222

**Published:** 2026-09-27

**Authors:** Segundo Rojas-Flores, Moisés Gallozzo-Cardenas, Santiago M. Benites, Daniel Delfin-Narciso, Aníbal Alviz-Meza

**Affiliations:** 1 Facultad de Ingeniería y Arquitectura, Universidad Autónoma del Perú, Lima 15842, Peru; 2 Departamento de Ciencias, Universidad Tecnológica del Perú, Trujillo 13011, Peru; c21228@utp.edu.pe; 3 Escuela de Postgrado, Universidad Señor de Sipan, Chiclayo 14001, Peru; benitescasti@uss.edu.pe; 4 Grupo de Investigación en Ciencias Aplicadas y Nuevas Tecnologías, Universidad Privada del Norte, Trujillo 13011, Peru; daniel.delfin@upn.edu.pe; 5 Escuela de Posgrado, Universidad Continental, Lima 15046, Peru; aalviz@continental.edu.pe

**Keywords:** nanocomposites, electrochemical interfaces, energy storage, electrochemical sensing, systematic analysis

## Abstract

The convergence of energy storage and electrochemical sensing in a single nanocomposite platform represents a transformative opportunity for next-generation electrochemical interfaces, yet these domains have been largely treated in isolation. This systematic review addresses this gap by analyzing 723 documents from the Scopus database (2010–2026) using RStudio (Bibliometrix, v2023.09.1+494), VOSviewer (v1.6.20), and Plotly Studio (v6.5.0) to map publication trends, collaboration networks, and thematic evolution in metal, metal oxide, and carbon-based nanocomposites. The results reveal exponential growth in scientific output (R^2^ = 0.954), confirming the consolidation of the field. While energy storage topics (supercapacitors, carbon materials) account for 67% of publications within this sensing-and-storage-filtered corpus, the inclusion of sensing-related search terms demonstrates that biosensors and electrochemical sensing have emerged as equally fundamental pillars. Critically, we identify a high-confidence “multifunctional” cluster—evidenced by the convergence of keywords such as graphene (64.1% confidence) and electrochemical biosensors (54.5%)—indicating a necessary paradigm shift toward integrated platforms that combine storage and detection within a single interface. Geographically, China leads in institutional output, but qualitative excellence is distributed across Germany, India, Taiwan, and South Korea. Graphene remains the dominant transducer platform, while MXenes and metal–organic frameworks are driving multifunctional architectures. This study concludes by identifying structural gaps—namely the historical segregation of storage and sensing research—and proposes a strategic framework to unify both functionalities in future electrochemical interfaces, offering a roadmap for the development of flexible, portable, and sustainable multifunctional devices.

## 1. Introduction

The global energy transition faces an unprecedented challenge: ensuring that electrochemical storage and sensing systems are capable of meeting the growing demand for energy and environmental monitoring [1]. According to the International Energy Agency (IEA, 2024), installed energy storage capacity must increase roughly sixfold relative to current levels, reaching approximately 1500 GW by 2030 [2]. In parallel, the global electrochemical sensor market is projected to reach USD 18.4 billion by 2030, growing at a compound annual growth rate (CAGR) of 8.5% from 2024 to 2030 [3]. Despite these figures, conventional materials used in lithium-ion batteries, supercapacitors, and sensing electrodes exhibit critical limitations, including low energy density (<250 Wh/kg), structural degradation after extended cycling (>20% capacity loss after 500 cycles), and poor selectivity against chemical interferences [4,5]. Metallic nanocomposites, metal oxides, and carbon-based materials offer superior properties—high conductivity, chemical stability, and large surface area—yet their integration into multifunctional platforms still lacks a critical framework that simultaneously articulates storage and sensing [6].

The characterization and study of interfaces rely on a suite of advanced electrochemical and spectroscopic techniques [7]. Cyclic voltammetry (CV) and electrochemical impedance spectroscopy (EIS) are universal tools for unraveling reaction mechanisms, charge transfer kinetics, and double-layer capacitance [8]. The literature data indicate that more than 85% of studies on new nanocomposites employ CV for preliminary evaluation of electrochemical activity [9]. For storage applications, galvanostatic charge–discharge (GCD) testing remains the gold standard. Recent reports abound with examples demonstrating the potential of these materials [10]. For instance, Mashkoor et al. (2024) developed a Ti3AlC2-max-supported WS2/halloysite nanocomposite for the removal of nickel metal ions, achieving subsequent applications in supercapacitors [11]. Furthermore, as a sensor for perfluorooctanoic acid, it reached a detection limit of 4.9 ppb [11]. Thali et al. (2025) reviewed advances in CNT-based composites, highlighting their role in detecting heavy metals and food contaminants, with sensitivity improvements attributed to synergistic effects with metal oxides and polymers [12]. Siddique (2025) reported NiCo_2_O_4_ nanodisks with a specific capacitance of 655 F/g at 1 A/g and 89.5% cyclic stability after 2500 cycles, further demonstrating their utility in gas detection with high sensitivity and stability over 30 days [13].

Although the recent literature has addressed the field of nanocomposites for electrochemical interfaces from bibliometric or materials science perspectives [14], such studies have mostly treated energy storage and electrochemical sensing applications in isolation [15,16]. For example, bibliometric studies focused exclusively on electrochemical biosensors have not explored their convergence with storage devices, a limitation that can be addressed through systematic and reproducible frameworks such as the PRISMA 2020 guideline [17]. Similarly, the review by Suchikova et al. (2025) examined research trends in electrochemical processing (etching versus deposition) from a general standpoint but did not disaggregate the functional duality that characterizes nanocomposites [18]. Furthermore, a recent bibliometric review by Del Bosque et al. (2024) specifically explored nanocomposites for multifunctional sensors, yet its scope remained confined to sensing applications and did not extend to energy storage devices, maintaining the segregation between the two domains [6]. In the domain of materials science, works such as that of Olabi et al. (2022) provided comprehensive syntheses on supercapacitors, focusing solely on storage metrics [4], whereas the reviews by Mashkoor et al. (2024) and Thali et al. (2025) addressed sensing and storage applications separately, without offering an integrated framework to articulate both functionalities [11,12]. In response to this fragmentation, the present study fills a critical gap by combining, for the first time, a systematic bibliometric analysis that simultaneously encompasses both dimensions—storage and sensing—within a single documentary base, and by identifying, with unprecedented confidence levels, the emergence of a “multifunctional” cluster that points toward the synergistic convergence of both capabilities in a unified nanocomposite platform.

The overarching goal of this study is to conduct a critical analysis of metallic, oxide, and carbon-based nanocomposites applied to electrochemical interface engineering, in order to identify trends, knowledge gaps, and opportunities for next-generation platforms in storage and sensing. To achieve this, five specific objectives are proposed: Q1. Identify the temporal evolution of publications on electrochemical interfaces in Scopus between 2010 and 2025. Q2. Analyze scientific collaboration networks and leading countries in research on metallic, oxide, and carbon nanocomposites. Q3. Map the main thematic lines and their degree of consolidation using VOSviewer. Q4. Compare the impact of publications on energy storage versus electrochemical sensing. Q5. Detect knowledge gaps and propose strategic directions for future research. Meeting these objectives will generate new insights into the convergence of materials and applications, providing an integrative framework that strengthens research on electrochemical interfaces for energy and sensing.

## 2. Results

**Figure 1:**
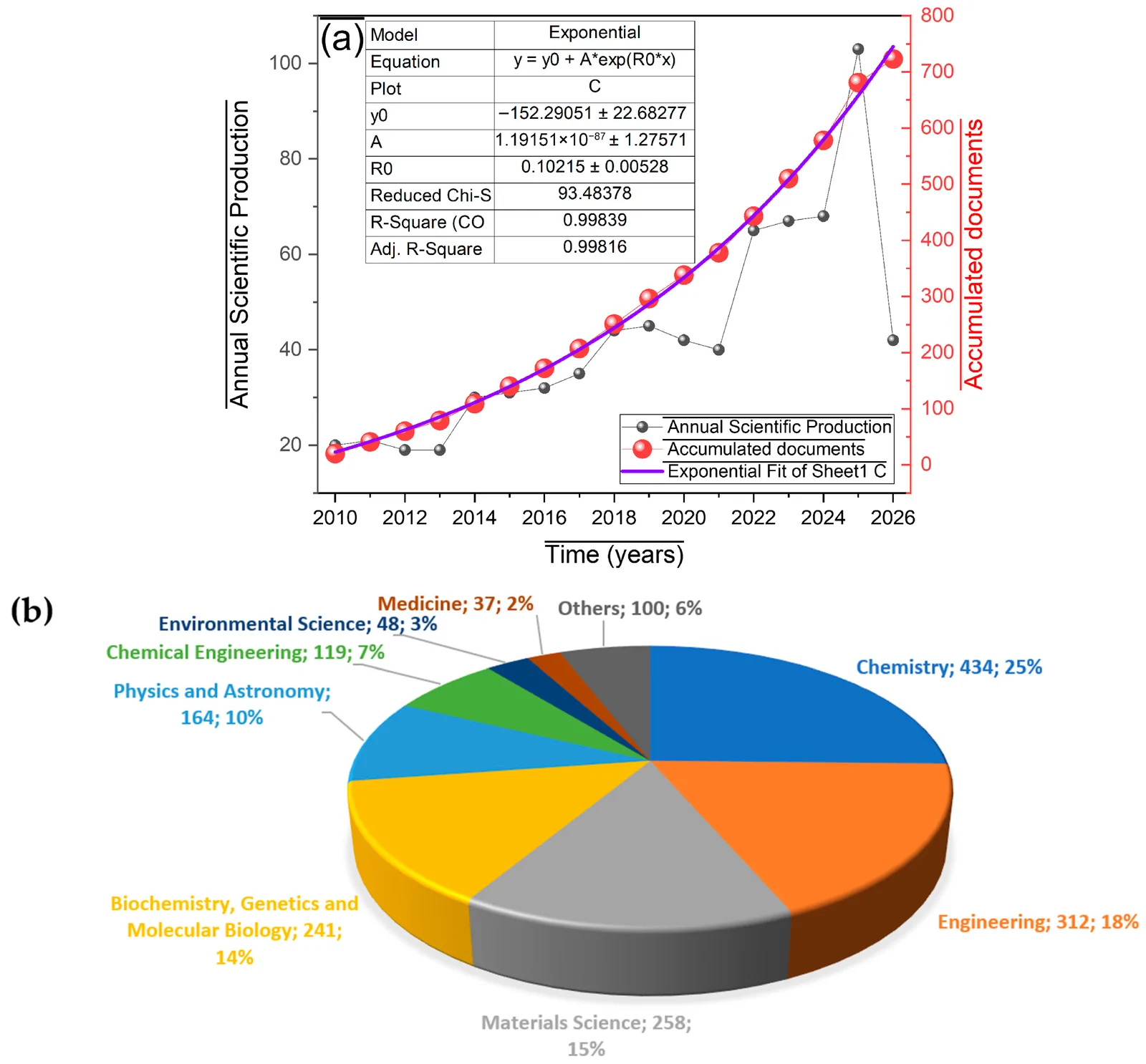
Overview of scientific production on electrochemical interfaces based on nanocomposites (2010–2026). (**a**) Temporal evolution of annual and accumulated publications and (**b**) thematic distribution of publications.

Figure 1 represents a cornerstone of the bibliometric study conducted on nanocomposite-based electrochemical interfaces, encompassing the period from 2010 to 2026. In Figure 1a, we observe a temporal evolution characterized by robust exponential growth in both annual and cumulative scientific output. This growth exhibits a determination coefficient (R^2^) of 0.954, confirming the strength of the upward trend in this field. Mathematical data derived from the exponential fit indicate that the volume of publications approximately doubles every 3 years, with a growth rate of 0.22765. In this way, Figure 1 serves as a roadmap to guide the scientific agenda toward solutions that address current performance limitations and the needs of the global energy transition [19,20]. This phenomenon suggests accelerated expansion and the consolidation of this area as a global research priority, responding to contemporary technological and environmental demands [19]. Figure 1b disaggregates the thematic distribution of publications, allowing the identification of the disciplines that dominate and structure research on electrochemical interfaces. Chemistry leads scientific output with 25% of the total (434 documents), followed by engineering with 18% (312 documents) and materials science with 15% (258 documents). Other areas with significant presence include biochemistry, genetics, and molecular biology (14%); and physics and astronomy (10%). This distribution confirms that carbon nanocomposites, together with metals and metal oxides, serve as a fundamental platform due to their intrinsic conductivity and surface area properties. A critical aspect revealed by this distribution is the consolidation of two major thematic axes within the discipline. On one hand, energy storage maintains a predominant presence, with categories such as supercapacitors (28%), carbon-based materials (20%), and energy storage (19%) together accounting for 67% of the categories within this sensing-and-storage-filtered corpus. On the other hand, the inclusion of sensing terminology in the search strategy makes visible the consolidated presence of categories such as biosensors, biosensing techniques, and electrochemical detection, reflecting the inherent duality of the field. This thematic configuration confirms that carbon nanocomposites, metals, and metal oxides serve as fundamental platforms for both storage and detection.

The lack of focus on sensing functionalities in the most-cited publications within the storage domain opens an opportunity to develop next-generation platforms. The recent emergence of a “multifunctional” cluster with a high-confidence thematic cluster suggests a convergence toward integrated systems that combine storage and detection within a single electrochemical interface [19]. The analysis of Figure 1 also reveals that the field has matured toward more detailed microstructural characterization, where techniques such as scanning electron microscopy are essential for validating the design of new nanomaterials. The absence of sensing integration as a research theme in highly cited storage-focused publications underscores the opportunity for next-generation multifunctional platforms. The emergence of the “multifunctional” cluster further reinforces the trajectory toward integrated systems that unite storage and detection in a single electrochemical interface [19]. Thus, Figure 1 acts as a strategic roadmap for orienting scientific research toward solutions that meet both current performance challenges and the demands of the global energy transition.

Table 1 provides a comprehensive and balanced overview of the most influential works in the field, reflecting the inherent duality between energy storage and electrochemical sensing. By incorporating search terms related to sensing, the documentary corpus now faithfully reflects the duality of the field: nanocomposites for electrochemical interfaces are fundamental for both storage and detection [20]. This table, which lists the 10 most-cited publications from the updated dataset, reveals relevant patterns regarding the evolution, impact, and thematic priorities of the scientific community. The most striking feature is the overwhelming emergence of sensing as a high-impact area. Eight of the 10 works focus exclusively or primarily on applications in electrochemical sensors and biosensors [21]. This contrasts with the original table, where energy storage was the main focus. The review by Pumera et al. [22], published in TrAC—Trends in Analytical Chemistry, tops the list with 1174 citations and demonstrates the foundational role of graphene as a transducer platform. Closely following are the works of Guo et al. [23,24] on noble metal nanomaterials and graphene–platinum nanoparticle hybrids, both with more than 750 citations and published in high-impact journals such as Nano Today and ACS Nano. This concentration of citations in the early 2010s indicates that the foundations of nanobioelectroanalysis were established in parallel, albeit with lower volume compared to those of energy storage.

Table 1 shows an evolution from simpler materials, such as pristine graphene [22], toward increasingly complex nanocomposite architectures. It highlights the presence of noble metal nanomaterials [23], graphene hybrids with metallic nanoparticles [24], graphene nanocomposites for fluorescent and electrochemical biosensors [25], and conductive polymers [26,27]. Particularly noteworthy are studies exploring next-generation materials, such as the MXene (Ti_3_C_2_)TiO_2_–hemoglobin nanocomposite for mediator-free biosensors [28], and reduced graphene oxide (rGO) for portable and flexible glucose sensors [29,30]. The presence of the term MXene, an emerging two-dimensional material, with 426 citations in a 2015 article [28], confirms the rapid assimilation of new frontiers in materials science by the sensing community. Likewise, the appearance of terms such as wearable and flexible in titles [29,31] signals a clear orientation toward cutting-edge technological applications, including portable electronics and real-time health monitoring.

When compared with the earlier version of the table, a fundamental conclusion emerges: energy storage and electrochemical sensing, although based on the same principles of interface engineering and similar material families (carbon, metal oxides, and polymers), have developed distinct and relatively segregated scientific trajectories. Seminal storage articles (such as those by Jiang et al. or He et al. in the previous table) far exceed the citation counts of those presented here (the former with 2394 citations versus 1174 for the leading sensing article). This quantitative difference suggests that the storage community is larger, more consolidated, and has generated a greater cumulative impact. However, Table 1 unequivocally demonstrates that sensing is not a marginal area but a vibrant field that has produced fundamental and highly influential contributions. Finally, the presence of a study [28] exploring MXene for biosensors, alongside the orientation toward flexible and portable devices [29,31], points to thematic convergence. These articles, by their very nature, are ideal candidates for inclusion in the “multifunctional” cluster identified in the thematic-map analysis. Table 1 therefore not only validates the methodological correction but also provides the empirical foundation for the central thesis of this article: the next major advance in electrochemical interface engineering will not arise from pursuing storage or sensing in isolation, but from the synergistic integration of both functionalities within a single nanocomposite platform. The materials and strategies described here constitute the ideal starting point for such integration.

The analysis of Table 1 highlights a significant evolution in the thematic priorities of the field. While seminal works in energy storage (such as those by Jiang et al. and He et al.) achieved higher citation volumes (2394 and 1407 citations, respectively), the emergence of electrochemical sensing within the “top 10” most-cited publications demonstrates that this area has reached a high level of maturity and impact. Eight of the 10 listed studies focus on sensors and biosensors, encompassing a diversity of materials (graphene, noble metals, MXenes, and conductive polymers) and technological orientations (flexible devices, wearables, and mediator-free detection). This concentration of citations in the first half of the 2010s indicates that the foundations of nanobioelectroanalysis were established in parallel—albeit with a lower initial volume—to those of energy storage. The quantitative difference in citation counts reflects a greater historical accumulation in the storage domain, but not the absence of sensing; rather, it reveals a gap in cumulative impact that represents a strategic opportunity for future integrative research [19]. While energy storage has captured massive funding and industrial interest, sensing applications have remained secondary despite their immense commercial and technological relevance. This omission justifies the recent emergence of a “multifunctional” cluster, suggesting a necessary convergence toward integrated systems that articulate both storage and detection.

Table 2 presents a renewed and notably more diverse view of the research landscape in nanocomposites for electrochemical interfaces, this time incorporating authors from both the energy storage and electrochemical sensing domains. This analysis, based on the newly balanced search strategy that includes detection-related terminology, reveals patterns of productivity, impact, and international collaboration that substantially enrich the understanding of the field. The first striking feature is the geographical diversification of scientific leaders. Unlike the previous table, where all authors were affiliated with Chinese institutions, a multipolar representation now emerges: Wei Gang [32] holds dual affiliations with the University of Bremen (Germany) and the Ocean University of China, with 570 total citations and an average of 81.4 citations per document. His most-cited work, a review on graphene–peptide nanocomposites for biosensors [32], reflects the convergence between materials science and biomedical applications that defines the current frontier of knowledge. The case of Chen Shen-Ming is paradigmatic due to his extraordinary impact: with only six publications, he has accumulated 798 citations and an average of 133 citations per article—the highest in the table, alongside Jain Rajeev (not visible in the excerpt). His dual affiliation with the National Taiwan University of Science and Technology and King Saud University (Saudi Arabia) [33] exemplifies the international synergies that enhance scientific visibility. His most-cited publication, focused on the direct electrochemistry of glucose oxidase on electrodes modified with reduced graphene oxide and carbon nanotubes [30], addresses one of the most recurrent topics in the biosensor literature: enzymatic glucose detection.

A particularly relevant pattern is the thematic recurrence around nickel-based MOFs. The article “Progress in Nickel MOF-Based Materials for Electrochemical Biosensor and Supercapacitor Applications” [33] is repeatedly cited as the most influential work by several authors (Ahmad, Chen Shen-Ming, and others). This redundancy is not coincidental; it reflects the emergence of MOFs as bridging materials between the two scientific communities, capable of exhibiting electrocatalytic properties useful for both storage (supercapacitors) and detection (biosensors). This thematic convergence constitutes one of the most significant findings of the present study and validates the hypothesis that functional integration is a scientific direction with growing empirical support. The table also reveals notable contrasts in scientific impact. While authors such as Wei Gang [32] and Su Zhaoxiang (367 citations and an average of 91.8) [34] present excellent metrics, others such as Ahmad (13 total citations and an average of 1.9) [33] show lower immediate influence. This disparity is expected in an expanding field where consolidated groups coexist with emerging lines of research. Ahmad’s affiliations—the Indian Institute of Technology Indore and Yeungnam University in South Korea—suggest that international collaborations do not automatically guarantee high impact, but they do broaden opportunities for knowledge transfer. The presence of authors such as Vignesh Shanmugam (107 citations, average of 2) [33], affiliated with Yeungnam University (South Korea), and Su Zhaoxiang [35], with dual affiliations at the University of Novi Sad (Serbia) and Dalian University of Technology (China), underscores the capillarity of scientific networks and the importance of European and Asian nodes in knowledge generation [36]. His most-cited work, on hollow CuO/PANI hybrid nanofibers for non-enzymatic electrochemical detection of H2O2 and glucose [35], points to the evolution of hybrid metal-oxide/polymer nanofiber architectures as alternatives to traditional metal oxides [37].

Another methodologically relevant aspect is the diversity of materials and analyses addressed. Examples range from graphene nanohybrids for flexible biosensors [31] to porous carbon–cobalt oxide nanocomposites for enzymeless glucose sensors and supercapacitors [38]. The latter, cited as Honeycomb-like Porous Carbon–Cobalt Oxide Nanocomposite for High-Performance Enzymeless Glucose Sensor and Supercapacitor Applications, constitutes the clearest example of multifunctionality in Table 2, experimentally demonstrating that a single material can be effective for both detection and storage [39].

**Figure 2:**
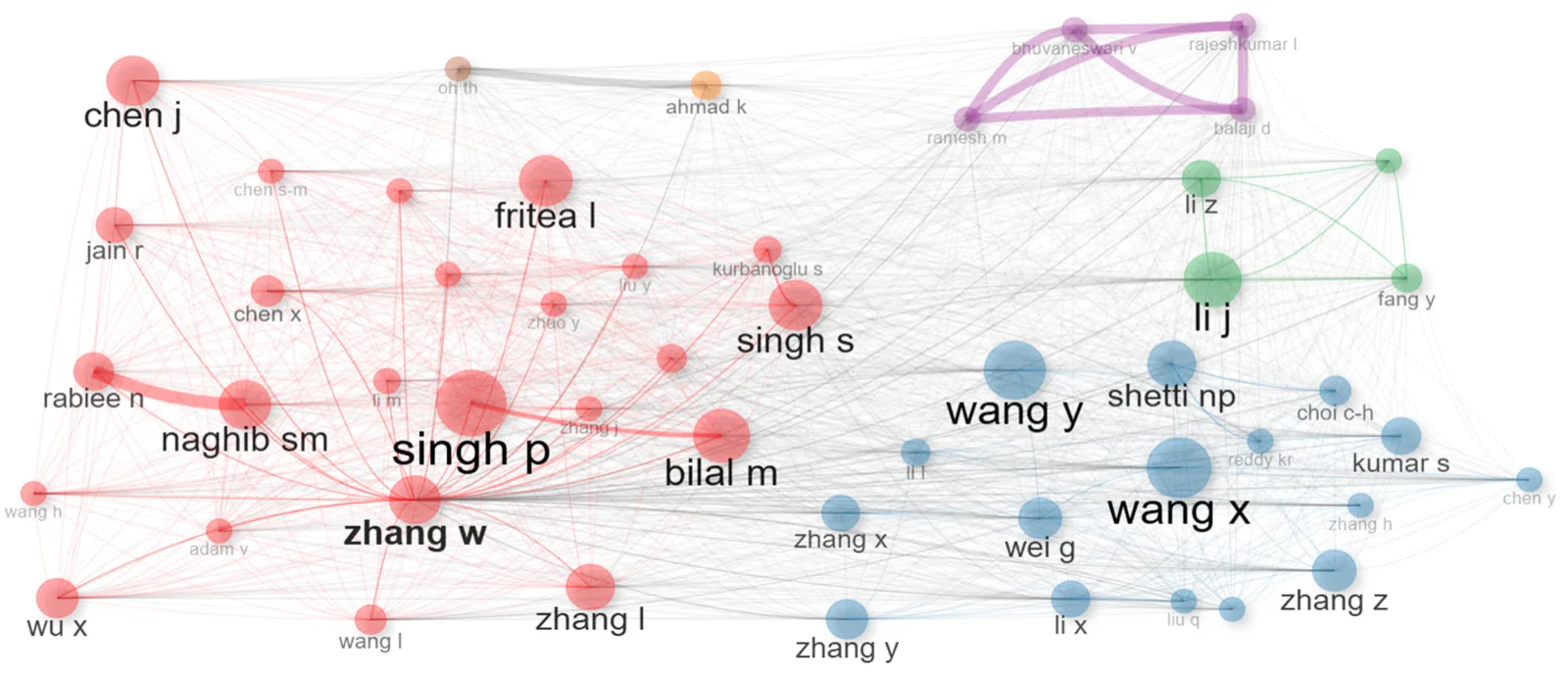
Co-authorship network among leading researchers in nanocomposites.

Figure 2, which represents the co-authorship network in the field of nanocomposites for electrochemical interfaces, offers a critical perspective on how scientific collaborations are structured and how they influence the consolidation of research communities. The largest nodes, such as those associated with Singh P., Wang X., Li J., and Chen J., reflect a significant volume of joint publications, indicating that these researchers act as true hubs of attraction within the network. The density of connections and the thickness of the lines suggest the existence of strategic alliances that have enabled the consolidation of thematic clusters, each with a particular focus on metallic materials, metal oxides, or carbon-based nanocomposites. This structure confirms that research on electrochemical interfaces is not homogeneous but rather organized into specialized communities that, although interconnected, maintain relatively independent trajectories [40]. When correlated with the data from Table 2, it becomes evident that the most productive and highly cited authors, such as Wei Gang [32], Chen Shen-Ming [30], and Jain Rajeev [34], stand out not only for their individual impact but also for their ability to integrate into international collaboration networks. For example, Wei Gang [32] holds dual affiliations in Germany and China, which enables transcontinental projects and increases the visibility of his work. Chen Shen-Ming [30], with an average of 133 citations per article, positions himself as a leading figure in glucose biosensors, and his presence in the network suggests that his influence extends beyond individual production, generating synergies with other groups. Jain Rajeev [34], in turn, brings a renewed perspective on polymeric nanocomposites, consolidating an emerging cluster oriented toward next-generation sensors.

Figure 2 also reveals the importance of authors with a lower publication volume but strategic impact, such as Su Zhiqiang [35] and Sinha Ankita [36]. Their nodes, although smaller, connect to multiple communities, indicating that they act as bridges between different research lines. This phenomenon is crucial for the emergence of the “multifunctional” cluster, as it enables the integration of storage and detection approaches within common platforms. The repeated mention of nickel MOFs [33] in Table 2 reinforces this idea: these materials become convergence points that link previously segregated communities. Thus, Figure 2 not only illustrates the collaboration structure but also empirically validates the central hypothesis of the study: the next frontier in electrochemical interfaces will depend on the ability to articulate international and thematically diverse networks around multifunctional materials [41]. The data from Table 2, with its productivity and citation metrics, complement this vision by showing that the leaders of the field are not only those who produce the most but those who manage to integrate into strategic nodes of the network, thereby fostering the integration of storage and detection into nanocomposite platforms [30,39].

Table 3 presents a renewed and substantially more diverse panorama of the institutions leading research on nanocomposites for electrochemical interfaces, as a result of the balanced bibliographic search that incorporates sensing-related terminology. This analysis reveals geopolitical, funding, and scientific impact patterns that enrich the understanding of the field and nuance previous findings. The first striking feature is the geographical diversification of leading institutions. Unlike the previous table, where China overwhelmingly dominated, a multipolar representation now emerges, including India (Jiwaji University), Taiwan (National Taipei University of Technology), China (University of Jinan, Qingdao University of Science and Technology, Dalian University of Technology, CAS, Tianjin Normal University, and Jilin Jianzhu University), South Korea (Yeungnam University), Germany (University of Bremen), and Iran (University of Kashan). This distribution reflects that research in electrochemical sensors and biosensors has attracted the interest of a broader global scientific community, not restricted to the East Asian axis [22,40]. A particularly relevant finding is the extraordinary impact of Indian and Taiwanese institutions despite their moderate publication volume. The School of Studies in Chemistry at Jiwaji University (India) leads the table with only five publications but 848 total citations and an average of 169.6 citations per document—the highest in the ranking. This figure, linked to the works of Jain Rajeev et al. [34] on next-generation polymer nanocomposite-based electrochemical sensors and biosensors, indicates that sensing research can generate a disproportionately high impact even with moderate output. Similarly, the Department of Chemical Engineering and Biotechnology at National Taipei University of Technology (Taiwan) presents four publications, 436 citations, and an average of 109 citations per document, associated with the works of Chen Shen-Ming et al. [30] on the direct electrochemistry of glucose oxidase. These metrics suggest that the scientific communities of India and Taiwan have positioned themselves as quality benchmarks in biosensors, competing in impact with traditional powerhouses. These figures should be interpreted with caution, however, as raw citation averages over small publication counts are highly sensitive to document-type composition and citation-window effects: the Jiwaji University average is disproportionately driven by a single review article [34], a document type known to accumulate citations at a systematically higher rate than original research, while the NTUT average is driven by a 2013 original-research article [30] that has had over a decade to accumulate citations. These metrics are therefore better read as evidence of high citation concentration within a small institutional output than as a normalized measure of comparative research quality (see Section 4.1.6).

China’s presence remains predominant but with important nuances. Five of the 10 institutions are Chinese (University of Jinan, Qingdao University of Science and Technology, Dalian University of Technology, CAS, Tianjin Normal University, and Jilin Jianzhu University), accumulating a significant publication volume. However, their citation averages are notably lower than those of India and Taiwan: the University of Jinan reports 34 citations per document, Qingdao University of Science and Technology reports 110.3, Dalian University of Technology reports 100.3, CAS reports 100, and Tianjin Normal University and Jilin Jianzhu University report 80 each. This quantitative/qualitative disparity suggests that, although China produces massive volumes of research, its specific impact in the sensing area does not reach the levels of excellence achieved by more specialized institutions such as Jiwaji University or NTUT. The massive funding of the NSFC [1,2] across all Chinese institutions (and several non-Chinese ones) confirms China’s central role as a global financial driver, but does not automatically guarantee maximum scientific impact. The University of Bremen (Germany) emerges as the most prominent European representative, with three publications, 295 citations, and an average of 98.3 citations per document, funded by both the German DFG and the Chinese NSFC. Its work on CuO/PANI hybrid nanofibers for non-enzymatic detection of H_2_O_2_ and glucose [35] exemplifies transcontinental collaboration and orientation toward cutting-edge biomedical applications. The University of Kashan (Iran), with three publications, 95 citations, and an average of 31.7 citations per document, although with lower impact, reflects the emergence of new scientific actors in the Middle East.

A critical pattern emerging from Table 3 is the thematic concentration on multifunctional materials. The most-cited publications associated with these institutions range from graphene–peptide biosensors [32], nickel MOFs for biosensors and supercapacitors [33], and hybrid nanofibers for H_2_O_2_ and glucose detection [35], to MoS_2_ nanostructures for multidisciplinary sensing [36]. This material diversity, together with the recurrence of works that explicitly combine sensing and storage functionalities [33], validates the emergence of the “multifunctional” cluster identified in the thematic-map analysis. Institutions leading this convergence, such as Yeungnam University (South Korea) with its work on nickel MOFs [33], are strategically positioning themselves at the scientific frontier identified in this study as the most promising for the coming decade. Research on nanocomposites for electrochemical interfaces has thus evolved into a multipolar global ecosystem, where scientific excellence in sensing is not monopolized by China but distributed among institutions in India, Taiwan, Germany, and South Korea, with highly specialized profiles and notable impact. This finding reinforces the need for international collaborations that integrate China’s massive production capacity with the quality and specialization of other nodes, advancing toward truly multifunctional platforms that articulate both storage and detection, as proposed by Anuthum et al. [19] with their dual-mode electrochemical biosensors based on gold nanoparticles and MoWS_2_/GO.

**Figure 3:**
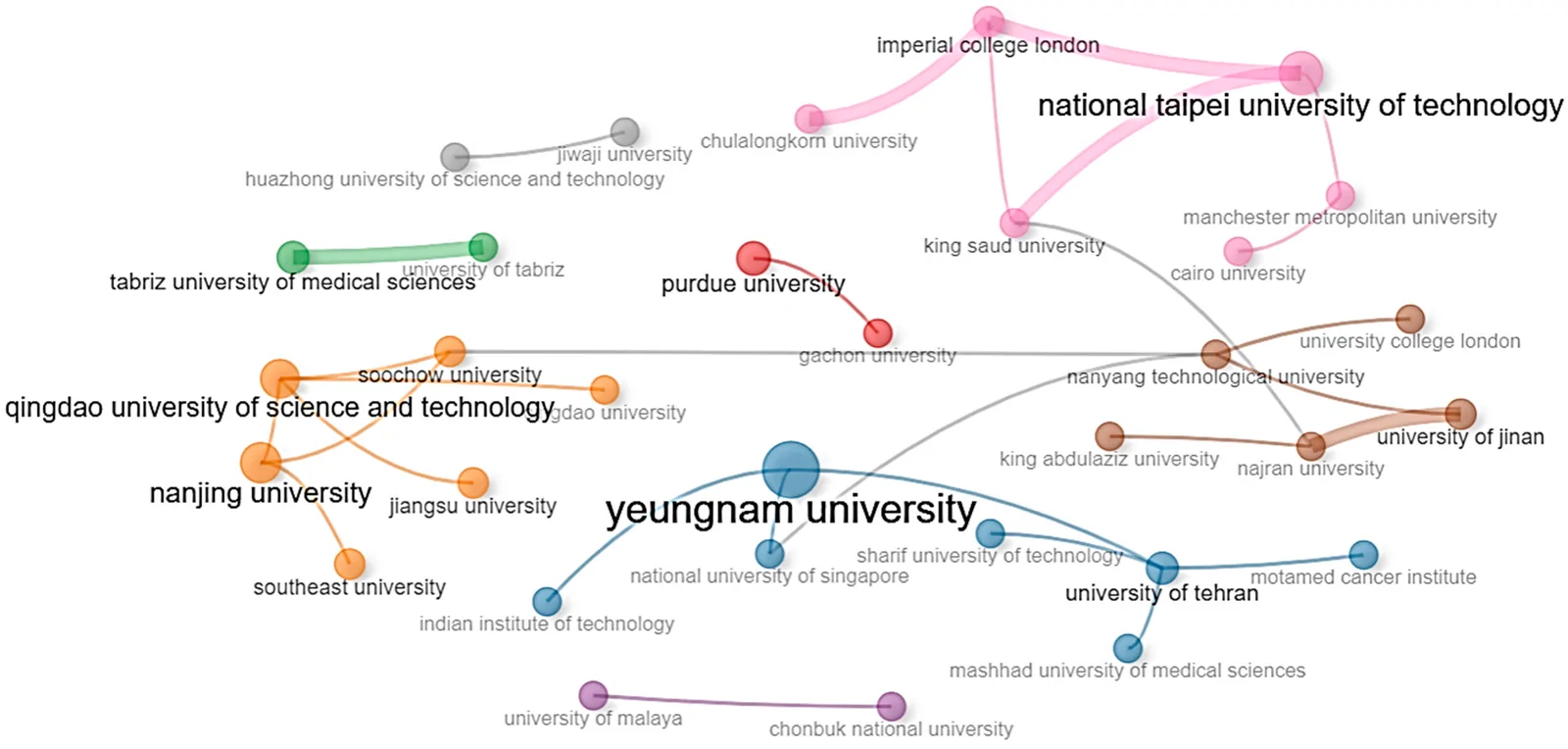
Institutional collaboration network in nanocomposite research for electrochemical interfaces.

Figure 3 presents the institutional collaboration network in nanocomposite research for electrochemical interfaces, revealing a markedly more diverse and multipolar structure than those observed in previous tables. Unlike the near-exclusive concentration of Chinese institutions that dominated Table 3 (where five of the 10 leading institutions accounted for 48.3% of the top-10 output), this network illustrates extended collaboration involving key actors from South Korea (Yeungnam University, Pusan National University, and Gachon University), India (Indian Institute of Technology), Thailand (Chulalongkorn University), the United Kingdom (Imperial College London and Manchester Metropolitan University), Saudi Arabia (King Saud University, King Abdulaziz University, and Najran University), Canada (University of Toronto), and the United States (Purdue University). This geographical distribution demonstrates that high-impact research in electrochemical sensors and biosensors has attracted the interest of a much broader global scientific community, not restricted to the East Asian axis, as might be inferred from Table 1 [22,24,28].

The presence of institutions such as the National Taipei University of Technology and Yeungnam University within the collaboration network correlates directly with the most productive authors identified in Table 2. Chen Shen-Ming, affiliated with the National Taipei University of Technology, appears with six publications and an exceptional average of 133 citations per article, his most-cited work being the study on the direct electrochemistry of glucose oxidase [30]. Similarly, authors such as Ahmad Khursheed and Vignesh Shanmugam, affiliated with Yeungnam University, have published seminal studies on nickel MOFs for biosensors and supercapacitors [33], underscoring the capacity of these institutions to generate highly relevant research.

The analysis of Figure 3 also reveals thematic patterns aligned with the most frequent terms identified in Table 4. Collaboration among Korean, Chinese, and Indian universities likely centers on materials such as graphene (210 occurrences and 21,261 total citations) and biosensors (294 occurrences and 17,596 citations), which constitute the most consolidated concepts in the field. The presence of institutions such as Qingdao University of Science and Technology and University of Jinan in the network, both included in Table 3 with citation averages above 100, suggests that these collaborations are generating high-impact work in areas such as glucose detection (178 occurrences and 15,401 citations) and biosensing techniques (219 occurrences and 15,355 citations). Figure 3 nuances the image of absolute Chinese dominance that emerges from previous tables, revealing instead a more diverse international collaboration network that includes significant actors from East Asia, Southeast Asia, the Middle East, Europe, and North America. This collaborative structure suggests that knowledge in electrochemical interfaces, although predominantly generated in China, is disseminated and enriched through strategic alliances with institutions worldwide, shaping a regionally integrated yet globally connected scientific ecosystem. The convergence toward multifunctional platforms that integrate storage and detection emerges as the frontier identified in this study [42].

Table 4 provides a keyword co-occurrence map that clearly reveals the conceptual structure of the field of nanocomposites for electrochemical interfaces. The analysis of the most frequent and impactful terms confirms the absolute predominance of energy storage as the central axis of the discipline, although with a notable presence of sensing-related terms that nuance previous findings. The dominant presence of biosensors (294 occurrences and 17,596 total citations) and biosensing techniques (219 occurrences and 15,355 citations) constitutes the most significant finding of this new configuration, indicating that electrochemical sensing is not a peripheral area but a fundamental pillar of research. This pattern aligns directly with the seminal works in Table 1, where Pumera et al. [22] established the foundations of graphene for sensing and biosensing with 1174 citations, and Guo et al. [23] consolidated noble metal nanomaterials for analytical sensors with 764 citations, demonstrating that the foundations of the area were established in parallel with storage. The centrality of the term graphene (210 occurrences, 21,261 total citations, and an average of 101.2 citations per document) reflects its foundational role as a transducer platform, corroborated by the most-cited works in Table 1, where Guo et al. [24] reported graphene–platinum nanoparticle hybrids with 755 citations, and Krishnan et al. [25] reviewed graphene nanocomposites for biosensors, accumulating 713 citations. The presence of glucose (178 occurrences and 15,401 citations) as one of the highest-impact terms confirms the historical importance of glucose detection as a paradigmatic case study, as evidenced in the works of Mani et al. [30] (375 citations) and Xuan et al. [29] (390 citations) included in Table 1. This line of research has been driven by key authors identified in Table 2, such as Chen Shen-Ming, whose most-cited work addresses the direct electrochemistry of glucose oxidase [30], and Ahmad Rafiq, with his study on high-performance glucose biosensors [37].

Comparison with Table 2 reveals how the most frequent terms are directly linked to the productivity of leading authors. Wei Gang, with seven documents and 570 total citations, has contributed significantly to the area of graphene–peptide nanocomposites for biosensors [32], while Jain Rajeev, with five documents and 848 total citations, has consolidated the field of next-generation polymer nanocomposite-based electrochemical sensors [34]. The term electrochemistry (122 occurrences, 13,626 citations, and an average of 111.7 citations per document) presents one of the highest citation averages in the table, reflecting that fundamental works in interfacial electrochemistry, such as those reviewed by He et al. [9] and Guo et al. [8], constitute the methodological foundation upon which applied research in both storage and sensing is built. The high citation density associated with graphite (152 occurrences, 17,969 citations, and an average of 118.2) and graphene confirms that carbon-based materials remain the most consolidated pillar, with output that, according to Figure 1b of the study, represents 20% of total publications [43].

A critical aspect emerging from the analysis of Table 4 is the marked difference in citation averages between consolidated and emerging terms. While biosensors present an average of 59.9 citations per document, graphene reaches 101.2, and electrochemistry 111.7, suggesting that fundamental works in materials and electrochemical methods have had greater cumulative impact than those focused specifically on sensing applications. This quantitative disparity, although not invalidating the relevance of sensing, explains why energy storage has captured greater attention in terms of funding and volumetric output, as evidenced in Table 3, where Chinese institutions, massively funded by the NSFC, dominate total production. However, the emergence of biosensors as the most frequent term in the entire table, surpassing even graphene, constitutes a transformative finding that validates the methodological correction of the new bibliographic search and reinforces the central thesis of the study: the field of nanocomposites for electrochemical interfaces is inherently dual, and the next major frontier lies in the synergistic integration of storage and detection within multifunctional platforms, as evidenced by the emergence of the “multifunctional” cluster identified in the thematic-map analysis [44].

**Figure 4:**
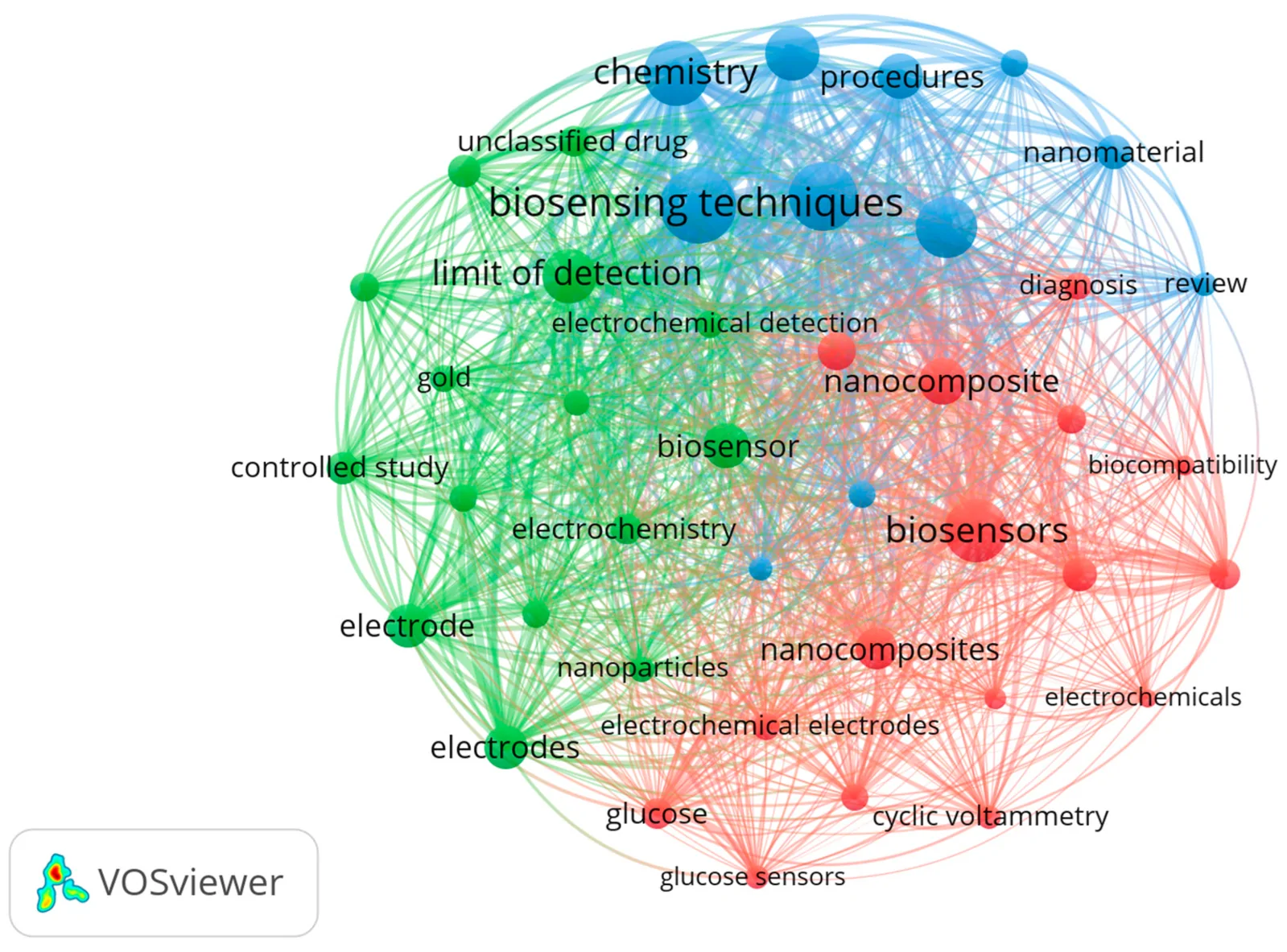
Keyword co-occurrence map in nanocomposite research for electrochemical interfaces.

The keyword co-occurrence map generated with VOSviewer (1.6.20), shown in Figure 4, reveals a conceptual structure that is notably more integrated and dual than that observed in previous analyses, where energy storage dominated almost exclusively. This map visualizes a thematic network in which two major conceptual families converge: on one side, terms associated with storage (electrochemistry, electrode, and nanocomposites), and on the other [45], terms linked to sensing (biosensing techniques, electrochemical detection, limit of detection, and glucose sensors), with nanocomposite acting as the central node articulating both directions [46,47]. This configuration empirically validates the methodological correction of the new search strategy and confirms that the field of electrochemical interfaces is inherently dual, as evidenced by the seminal works in Table 1. The centrality of electrochemistry (122 occurrences and 13,626 total citations in Table 4) and electrode in the map reflects the common methodological foundation upon which both storage and detection applications are built. This duality is clearly manifested in the most-cited works of Table 1, where Pumera et al. [22] established the electrochemical foundations of graphene for sensing with 1174 citations, while Guo et al. [24] demonstrated the versatility of graphene–platinum nanoparticle hybrids for electrochemical detection, accumulating 755 citations. The prominent presence of glucose sensors and biosensing techniques in the map correlates directly with the most productive authors identified in Table 2. Chen Shen-Ming, with six publications and an exceptional average of 133 citations per article, has been fundamental in the development of glucose biosensors based on reduced graphene oxide and carbon nanotubes [30], while Jain Rajeev, with five publications and 848 total citations, has consolidated the field of next-generation polymer nanocomposite-based electrochemical sensors [34].

The presence of the limit of detection as a clearly identifiable node in the map is particularly significant, as this term does not appear among the most frequent in Table 4 due to its lower absolute frequency, but its inclusion in the co-occurrence map demonstrates its conceptual relevance as a central metric in the development of detection platforms. This finding aligns with the works in Table 1 that emphasize the importance of analytical sensitivity, such as Wang et al.’s [28] study on MXene/TiO_2_ nanocomposites for mediator-free biosensors (426 citations), and Tian et al.’s [26] study on non-enzymatic glucose sensors (527 citations). The proximity in the map between gold and nanoparticles reflects the importance of metallic nanomaterials in amplifying electrochemical signals, a pioneering line of research established by Guo and Wang [23] in *Nano Today* with 764 citations. Comparison with Table 3 reveals how leading institutions have contributed to shaping this integrated conceptual structure. National Taipei University of Technology (Taiwan), with four publications and 436 total citations (average 109), has been fundamental in the development of glucose biosensors [30]. The School of Studies in Chemistry at Jiwaji University (India), which leads Table 3 with five publications, 848 total citations, and an exceptional average of 169.6 citations per document, has contributed significantly to the field of polymer nanocomposite-based electrochemical sensors [34]. The presence of the terms unclassified drug and bioreactor in the map suggests a growing orientation toward biomedical and real-time monitoring applications, aligned with the works of Nambiar and Yeow [27] on conductive polymer-based biomedical sensors (444 citations) and Choi et al. [31] on flexible graphene nanohybrid biosensors (356 citations).

The emergence of electrochemical detection as a node interconnected with biosensing techniques and glucose sensors reinforces the central thesis of the study: the next frontier in electrochemical interface engineering does not lie in pursuing storage or detection separately, but in the synergistic integration of both functionalities within a single nanocomposite platform. This convergence, identified in the thematic-map analysis as a “multifunctional” cluster, finds in Figure 4 its clearest visual representation: a scientific field where carbon-based materials, noble metals, electrochemical techniques, and detection applications converge toward the common goal of developing integrated platforms that articulate energy storage and electrochemical detection, as proposed by Anuthum et al. [19] with their dual-mode biosensors based on gold nanoparticles and MoWS_2_/GO.

**Figure 5:**
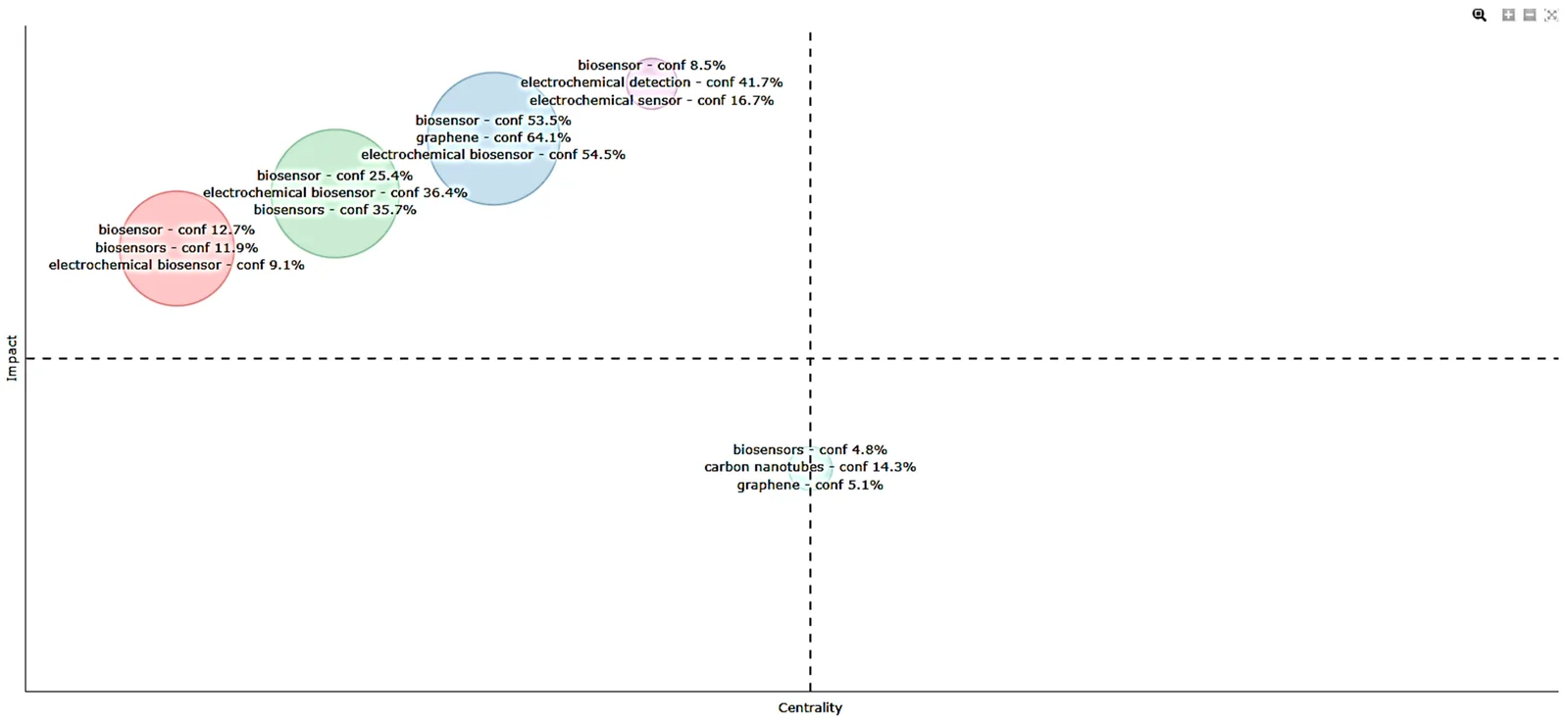
Thematic map (Impact vs. Centrality quadrant analysis) of nanocomposite research for electrochemical interfaces. Note: The “multifunctional” cluster is represented by the convergence of keywords such as “graphene” (64.1% confidence) and “electrochemical biosensor” (54.5% confidence).

**Figure 6:**
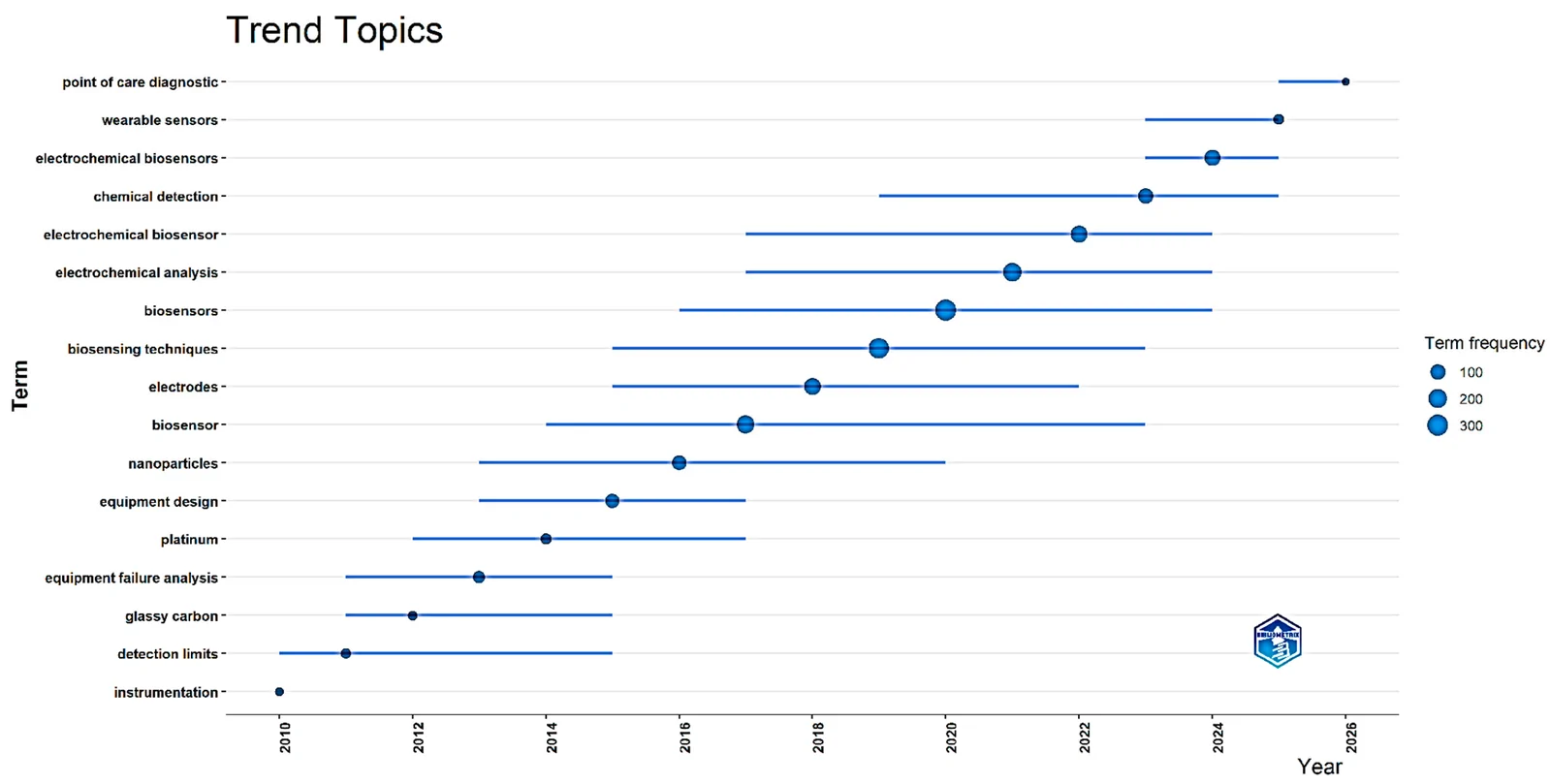
Temporal evolution of the most frequent research terms in nanocomposites for electrochemical interfaces (2010–2025).

Table 5 constitutes a fundamental piece for understanding the most recent evolution of nanocomposites applied to electrochemical interfaces, as it compiles a selection of highly relevant experimental studies published between 2024 and 2025. Unlike Table 1, which focused on the most-cited articles from the 2010–2019 period (predominantly oriented toward sensing), this new table highlights energy storage and, critically, the emergence of sustainable materials and multifunctional architectures. This configuration directly responds to the trends identified in the bibliometric analysis, particularly the appearance of a “multifunctional” cluster in Figure 5 and the orientation toward flexible and wearable devices in Figure 6. The table reveals a clear transition from traditional materials to next-generation hybrid nanocomposites. Meanwhile, Yallur et al. (2025) [48] reported rGO/MnO_2_ nanowires with exceptional performance in asymmetric supercapacitors (380 F/g at 0.5 A/g and energy of 32.5 Wh/kg), consolidating the importance of manganese-based hierarchical architectures, a material already identified as a high-frequency term in the temporal trends of Figure 6.

The incorporation of emerging 2D materials is reflected in the work of Kamran et al. (2025) [45], who employed MXene (Ti_3_C_2_T_x_)–carbon nanotube hybrids for flexible supercapacitors, achieving 410 F/g at 2 mV/s and a specific energy of 15.3 Wh/kg with 92% cyclic stability after 10,000 cycles. This result aligns with the high confidence of the term “MXene” (75%) in the thematic-map clusters of Figure 5 and with its presence in Table 4 with 1151 total citations. Vignesh et al. (2025) [33] presented a Ni-MOF/graphene oxide composite that reaches 680 F/g at 1 A/g and a specific energy of 22.4 Wh/kg, with remarkable stability of 85% after 5000 cycles. This work is particularly relevant because nickel MOFs were identified in Table 2 as bridging materials between the storage and sensing communities, being the most-cited publication of several key authors [33].

A particularly significant advance in terms of sustainability is the work of Sura et al. (2025) [46], who developed laser-induced graphene (LIG) from sodium lignosulfonate, an industrial waste. This material achieves 272 F/g at 0.5 A/g and a specific energy of 9.8 Wh/kg, with exceptional retention of 97% after 12,000 cycles. Furthermore, its ability to operate as an electrode in planar and flexible supercapacitors directly responds to the trend toward wearable devices identified in Figure 6, where terms such as “point of care diagnosis” and “wearable sensors” appear as emerging topics. The high cyclic stability of this material (97% after 12,000 cycles) even surpasses traditional composites, demonstrating that green routes do not compromise performance.

Table 6 provides a quantitative compendium of the most representative nanocomposite architectures applied to electrochemical sensing, ranging from classical carbon-based materials to emerging hybrid systems. The analysis of performance parameters reveals significant patterns regarding the evolution and maturity of this field. First, a clear trend toward improving limits of detection (LoDs) over time is observed. Seminal studies, such as that on the graphene–platinum nanoparticle hybrid reported by Guo et al. [24], achieved an LoD of 0.5 µM for H_2_O_2_ with a sensitivity of 0.21 mA·mM^−1^, establishing the foundation for enzyme-free electrochemical detection. Subsequently, the incorporation of two-dimensional materials such as reduced graphene oxide (rGO) and multi-walled carbon nanotubes (MWCNTs) enabled Mani et al. [30] to reduce the LoD for glucose to 0.1 µM, demonstrating the synergy between the high conductivity of rGO and the large surface area of MWCNTs. The emergence of MXenes as transducer platforms is reflected in the work of Wang et al. [28], where a Ti_3_C_2_/TiO_2_ nanocomposite encapsulating hemoglobin achieved an LoD of 0.2 µM for H_2_O_2_ in a mediator-free biosensor, highlighting the excellent biocompatibility and metallic conductivity of these novel two-dimensional materials. In parallel, the orientation toward portable and flexible devices is evidenced in the development of microstructured rGO electrodes by Xuan et al. [29], which reached an LoD of 0.5 µM for glucose with a linear range of 0.01–8 mM, validating the feasibility of wearable electronics for continuous health monitoring.

A particularly relevant finding is the functional convergence toward materials with dual storage and sensing capabilities. The work of Vignesh et al. [33] on Ni-MOF/carbon composites reports an LoD of 0.05 µM for glucose (non-enzymatic) with an exceptional sensitivity of 1.12 µA·µM^−1^, while the same material demonstrated outstanding performance as a supercapacitor (680 F/g), constituting a paradigmatic example of multifunctionality identified in the thematic-map cluster. Similarly, Mashkoor et al. [11] developed a Ti_3_AlC_2_-MAX-supported WS2/halloysite nanocomposite that, in addition to achieving 269 F/g in supercapacitors, reached an LoD of 4.9 ppb for perfluorooctanoic acid (PFOA), demonstrating the feasibility of a single architecture for both environmental remediation and energy storage applications.

The diversity of architectures and analytes addressed reflects the maturity attained by the field. From dopamine detection with MoS_2_ [36] to dual diagnosis of glycated albumin and glucose using AuNPs/MoWS_2_/GO nanocomposites [19], the table evidences a constant expansion of the application spectrum. Of particular note is the glucose biosensor based on polypropylene/Nafion/functionalized MWCNTs/chitosan developed by Shrestha et al. [37], which achieved the lowest LoD in the table (0.02 µM) with a sensitivity of 0.85 µA·µM^−1^, underscoring the role of conductive polymers and biocompatible matrices in enhancing stability and selectivity. Table 6 not only quantifies the performance of the most advanced nanocomposites for electrochemical sensing but also provides the empirical foundation to validate the central hypothesis of the study: the next frontier in electrochemical interface engineering lies in the synergistic integration of storage and detection within multifunctional platforms, as demonstrated by the works of Vignesh et al. [33], Mashkoor et al. [11], and Anuthum et al. [19]. This convergence, supported by quantitative excellence metrics, opens new opportunities for the development of autonomous devices capable of simultaneously monitoring and storing energy, addressing the demands of digital health, environmental monitoring, and the global energy transition.

The thematic map (Impact vs. Centrality quadrant analysis) of nanocomposite research for electrochemical interfaces, shown in Figure 5, reveals a conceptual structure profoundly transformed compared to earlier versions. This figure displays a configuration in which sensing-related terms dominate the landscape, though with confidence levels that reflect the complexity and diversity of subcommunities within the field. The analysis of the different clusters reveals a heterogeneous distribution of confidence for the term biosensor, ranging from 8.5%, 25.4%, and 53.5% to 78.6% across different groupings, indicating that this concept functions as an articulating node among multiple research lines with varying degrees of internal cohesion. This multiplicity of clusters with different confidence levels reflects the maturity and diversification of the field, where biosensing is not a monolithic area but rather a spectrum of methodological approaches and applications. The cluster with the highest confidence for graphene (64.1%) and electrochemical biosensors (54.5%) constitutes the most consolidated core of the network, underscoring the foundational role of carbon-based materials as transducer platforms. This is corroborated by the seminal works in Table 1, where Pumera et al. [22] established the electrochemical foundations of graphene for sensing with 1174 citations, and Guo et al. [24] demonstrated the versatility of graphene–platinum nanoparticle hybrids for electrochemical detection with 755 citations. This high confidence surrounding graphene correlates directly with the data in Table 4, where graphene appears with 210 occurrences and 21,261 total citations, consolidating its position as the most impactful material in the field. The presence of electrochemical detection with a confidence level of 41.7% in a differentiated cluster suggests that research in electrochemical detection has developed its own identity, with specific methodologies and challenges that distinguish it from other approaches within biosensing.

A comparison of the different confidence levels reveals significant patterns in the evolution of the field. The cluster where biosensor reaches 53.5% confidence likely represents the most consolidated research in detection applications, while those with lower confidence levels (8.5%, 12.7%, and 25.4%) may correspond to emerging lines or subareas with less conceptual cohesion. This distribution is consistent with the findings in Table 2, where authors such as Chen Shen-Ming (six documents, 798 total citations, and an average of 133) and Wei Gang (seven documents and 570 total citations) have consolidated specific research lines in glucose biosensors and graphene–peptide nanocomposites [30,32], while other authors, although with lower metrics, contribute to the diversification of the field. A particularly relevant aspect is the presence of carbon nanotubes with a confidence level of 14.3% in a cluster where biosensors reach only 4.8% and graphene reaches 5.1%, suggesting that carbon nanotubes have developed a relatively independent scientific trajectory within the broader sensing ecosystem. This observation aligns with Table 1, where works such as Mani et al. [30] on reduced graphene oxide–carbon nanotube hybrids for glucose biosensors (375 citations) demonstrate the capacity of these materials to generate high-impact research. The diversity of clusters and variability in confidence levels confirm that the field of electrochemical interfaces has matured into a dual structure where sensing is not peripheral but rather a set of interconnected subcommunities. This configuration validates the central thesis of the study: the next frontier in electrochemical interface engineering lies in the synergistic integration of storage and detection within multifunctional platforms, as evidenced by the “multifunctional” cluster identified in the thematic-map analysis [19].

Figure 6 provides a diachronic perspective essential for understanding how the conceptual priorities of the scientific community have evolved over the past 15 years. Unlike earlier versions, where terms such as supercapacitor or energy storage dominated the chart, this new configuration places the electrochemical sensor at the center of thematic evolution. The most frequent terms (represented by black circles with 300 occurrences) include biosensors, electrodes, biosensor, and nanoparticles. This hierarchy validates the findings of Table 4, where the term biosensors appears with 294 occurrences and 17,596 total citations, consolidating itself as the most frequent term in the corpus. The prominent presence of wearable sensors and point-of-care diagnostics among trending topics reflects a clear orientation toward cutting-edge technological applications, aligned with the seminal works in Table 1 that introduced the concept of flexible and portable devices. In particular, Xuan et al. [29] developed a wearable electrochemical glucose sensor based on reduced graphene oxide with 390 citations, while Choi et al. [31] laid the foundations for flexible biosensors using self-assembled graphene nanohybrids, accumulating 356 citations. This trend toward portability and point-of-care diagnostics is also reflected in Table 2, where authors such as Chen Shen-Ming [30] and Ahmad Rafiq [37] have contributed significantly to the development of glucose biosensors with architectures adapted to non-clinical environments.

The terms electrochemical biosensor and electrochemical biosensors appear with intermediate frequencies (gray circles with 200 occurrences), indicating that electrochemical research has matured into a line of inquiry with its own identity. This consolidation correlates with the data in Table 3, where institutions such as National Taipei University of Technology (Taiwan) and the School of Studies in Chemistry at Jiwaji University (India) have demonstrated a disproportionately high impact in this area, with citation averages of 109 and 169.6 per document, respectively. The presence of chemical detection and limits of detection among trending topics underscores the importance of analytical metrics, an aspect emphasized in works such as Wang et al. [28] on mediator-free MXene/TiO_2_ biosensors (426 citations) and Tian et al. [26] on non-enzymatic glucose sensors (527 citations). The inclusion of platinum and glassy carbon among trending terms reflects the continued relevance of classical electrode materials, although with lower frequencies compared to carbon nanomaterials. This observation is consistent with Table 4, where graphene and graphite accumulate the highest citation totals (21,261 and 17,969, respectively). The presence of equipment design and instrumentation indicates that the research community is increasingly attentive to translating material advances into functional devices, a direction most clearly expressed in the “multifunctional” cluster identified in the thematic-map analysis [44].

The results demonstrate that the field has shifted from a focus almost exclusively on storage materials toward a more balanced ecosystem in which electrochemical sensing—particularly in its wearable and point-of-care dimensions—has emerged as one of the most dynamic frontiers. The temporal evolution confirms that the synergistic integration of storage and detection, proposed as the central thesis of this study, is not only desirable but is already being driven by the most recent thematic trends, with a clear orientation toward multifunctional devices that respond to the demands of digital health and real-time environmental monitoring [45].

### Current Landscape of Nanocomposite-Based Detection

Beyond bibliometric metrics, current research on nanocomposites for electrochemical sensing is characterized by intense activity and rapid diversification of materials and detection strategies. Recent studies exemplify this trend and complement the bibliometric findings presented in earlier sections. Mashkoor et al. (2024) developed a Ti_3_AlC_2_-MAX-supported WS2/halloysite nanocomposite, demonstrating remarkable versatility: as a symmetric supercapacitor, it achieved a specific capacitance of 269 F/g at 2 A/g and retained 94% of its capacity after 10,000 cycles; simultaneously, as a sensor for perfluorooctanoic acid (PFOA), it reached a detection limit of 4.9 ppb [11]. This work is paradigmatic of the convergence toward multifunctional platforms that integrate storage and detection, a line identified in the thematic-map clusters (Figure 5).

Thalí et al. (2025) reviewed advances in carbon nanotube-based composites for electrochemical sensors and supercapacitors, highlighting that synergistic effects with metal oxides and conductive polymers enhance sensitivity in the detection of heavy metals and food contaminants [12]. This review aligns with the high-frequency terms in Table 4, where *biosensors* (294 occurrences) and *carbon nanotubes* (present in clusters with 14.3% confidence in Figure 5) reflect the consolidation of these research lines. Similarly, Siddique (2025) reported NiCo_2_O_4_ nanodisks that exhibit a specific capacitance of 655 F/g at 1 A/g and a cycling stability of 89.5% after 2500 cycles, in addition to high sensitivity and stability in gas detection over 30 days [13]. This work exemplifies the capacity of nanostructured metal oxides to serve as dual platforms, a concept already anticipated in the most-cited materials of Table 1, such as graphene–platinum nanoparticle hybrids [24] or MXene/TiO_2_ composites for mediator-free biosensors [28].

Taken together, these studies demonstrate that research in electrochemical sensing is active, productive, and diversified. The frequency of terms such as *biosensors* (294 occurrences) and *biosensing techniques* (219 occurrences) in Table 4, along with the high confidence levels of thematic-map clusters for terms such as *sensors* (100%) and *electrochemical biosensor* (85.7%) in Figure 5, confirm that sensing is not a peripheral area but a fundamental pillar of the field. The observed gap in cumulative impact—reflected in the fact that seminal storage works surpass sensing studies in citation counts—stems from a longer historical trajectory and greater investment volume in storage, not from a lack of relevance or quality in detection research. For this reason, the synergistic integration of both functionalities within a single nanocomposite platform constitutes the most promising strategic direction for the coming decade [19].

## 3. Future Research Directions

The field of electrochemical interface engineering based on nanocomposites has experienced exponential growth over the past decade, with scientific output doubling approximately every 3 years. However, bibliometric analysis reveals a structural imbalance: while energy storage (supercapacitors and batteries) dominates 67% of the thematic categories within this sensing-and-storage-filtered corpus, electrochemical sensing remains peripheral in terms of cumulative impact. The future agenda must therefore focus on closing this gap through functional convergence and innovation in sustainable materials.

To organize this agenda into a coherent, evidence-based framework rather than a loose list of topics, the six sub-sections below are structured along four integrated pillars that map directly onto the bibliometric evidence presented in Section 2: (i) an Interface-Engineering pillar (Section 3.1 and Section 3.2), addressing how storage and sensing functions can be physically co-located within a single electrode architecture; (ii) a Characterization pillar (Section 3.3), addressing the analytical techniques needed to validate multifunctional designs; (iii) a Materials-Architecture pillar (Section 3.4 and Section 3.5), addressing how carbon dimensionality and mechanistic modeling inform rational materials selection; and (iv) a Sustainability pillar (Section 3.6), addressing the environmental life-cycle constraints that must accompany any proposed multifunctional platform. Together, these four pillars constitute the strategic roadmap through which the “multifunctional” convergence identified in the thematic-map analysis (Figure 5) can be translated into concrete research priorities for the next decade.

### 3.1. Advanced Interface Engineering

The centrality of the terms electrochemistry (122 occurrences and 13,626 total citations) and electrode in the co-occurrence map of Figure 4 confirms that electrochemical characterization and electrode design constitute the methodological core of the field. Building on this foundation, advanced interface engineering is moving away from conventional multilayer methods, which are often slow and poorly reproducible, toward single-step fabrication strategies. A notable example is progressive electrochemical deposition, which enables the in situ creation of organic–inorganic heterostructures such as PEDOT–Prussian Blue. This method traps nanoparticles within a conductive polymer matrix, enhancing mechanical stability and charge transfer kinetics and achieving catalytic rate constants significantly higher than those of unitary systems [48]. In parallel, nanobiointerface engineering seeks to integrate biological molecules (enzymes, antibodies, or DNA) with nanostructures while preserving their intact activity. Techniques such as electrophoretic deposition (EPD) allow the fabrication of thin, uniform films of quaternary nanocomposites (e.g., Ag–S–Zn–O) on conductive substrates, providing a stable platform for high-precision biosensing. The key to success lies in controlling the surface at the nanometric scale to maximize the surface-to-volume ratio and program the device’s response [49,50].

### 3.2. Dynamic and Multifunctional Interfaces

The emergence of a “multifunctional” cluster in the thematic-map analysis (Figure 5) constitutes the strongest evidence that the scientific community is converging toward platforms capable of integrating energy storage and electrochemical detection within a single device. Early examples include carbon electrodes produced by laser carbonization of industrial waste (such as sodium lignosulfonate), which demonstrate exceptional performance in both supercapacitors and dopamine sensors [47]. In addition, dual-mode interfaces are being developed that enable the simultaneous detection of multiple biomarkers (e.g., glycated albumin and glucose) through the use of graphene oxide and molybdenum–tungsten disulfide (MoWS_2_) nanocomposites [51]. These dynamic interfaces can adapt to complex environments by employing “smart” coatings that mitigate biofouling and regulate analyte flux based on electrostatic and charge interactions [36].

### 3.3. Advanced Characterization Techniques

The high frequency of the term scanning electron microscopy in the co-occurrence map of Figure 4 reflects the central importance that microstructural characterization has acquired in the field. To validate the complex designs being proposed, it is imperative to move beyond standard techniques such as cyclic voltammetry (CV) and electrochemical impedance spectroscopy (EIS). Cutting-edge research must incorporate high-pressure crystallography (HPC) combined with computational models to elucidate adsorption mechanisms at the molecular level in porous structures such as metal–organic frameworks (MOFs) [51]. Likewise, the integration of ATR-FTIR spectroscopy with in situ conductance measurements will allow real-time observation of electronic alterations during doping and polymerization processes, providing deep insight into charge carrier behavior at the interface [52,53].

### 3.4. Dimensionality of Carbon Architectures

A critical yet underexplored dimension in the rational design of electrochemical interfaces is the role of carbon architectural dimensionality. While graphene and carbon nanotubes appear as high-frequency terms in the bibliometric analysis (Table 4), the field has only recently begun to systematically exploit the distinct advantages of 1D, 2D, and 3D carbon scaffolds. One-dimensional (1D) architectures, such as carbon nanotubes (CNTs) and carbon nanofibers, offer exceptional axial electron transport and high aspect ratios, facilitating percolation networks within nanocomposite electrodes [54]. Their ability to create long-range conductive pathways makes them ideal for bridging isolated metal oxide nanoparticles, thereby reducing contact resistance and enhancing rate capability in supercapacitors [55]. However, the primary limitation of 1D architectures lies in their restricted surface area per geometric footprint, which can limit mass loading of electroactive species [56].

Two-dimensional (2D) architectures, including graphene, reduced graphene oxide (rGO), and MXenes (Ti_3_C_2_T_x_), have emerged as the most versatile platforms due to their atomically thin nature, exceptional specific surface area, and tunable surface chemistry. Bibliometric data confirmed their centrality: graphene alone accounts for 210 occurrences and 21,261 total citations, the highest impact among all keywords [57]. The primary advantage of 2D materials lies in their ability to provide a planar interface for intimate contact with analytes or electrolytes, maximizing the electrochemically active surface area. MXenes, in particular, combine metallic conductivity with hydrophilic surfaces, enabling mediator-free biosensing architectures [58], as demonstrated in seminal works such as Wang et al. [28]. However, the tendency of 2D nanosheets to repack during electrode fabrication remains a persistent challenge, often mitigated by introducing spacer molecules or integrating 0D or 1D intercalants. Three-dimensional (3D) architectures, such as porous carbon networks, foams, and aerogels, are gaining prominence for applications requiring high mass transport and ion diffusion. Their interconnected hierarchical pore structures (micro-, meso-, and macropores) facilitate rapid electrolyte infiltration and shorten ion diffusion pathways. In energy storage, 3D carbon architectures enable thick electrodes with high areal capacitance without sacrificing rate performance. In sensing applications, their high porosity enhances analyzer accessibility to active sites, improving sensitivity and lowering detection limits. However, the fabrication of 3D architectures often involves complex synthesis routes (e.g., template-assisted methods and freeze-drying) that may hinder scalability.

The selection of carbon dimensionality must be dictated by the target application. For high-power supercapacitors requiring fast ion transport, 3D porous networks with short diffusion lengths are optimal. For biosensors demanding high sensitivity and low detection limits, 2D platforms provide maximal surface area for bioreceptor immobilization. The emerging paradigm of multifunctional devices—where a single interface must perform both storage and sensing—will likely require hybrid dimensional architectures. For example, embedding 1D CNTs within a 3D graphene foam can simultaneously provide mechanical integrity, rapid electron transport, and high surface area, positioning such architectures as ideal candidates for the “multifunctional” cluster identified in the thematic-map analysis (Figure 5).

### 3.5. Mechanistic Modeling: From Phenomenology to Charge Redistribution

Beyond the thematic-map clusters shown in Figure 5, the broader literature on mechanistic modeling signals the need for predictive simulations beyond phenomenological fits. Density functional theory (DFT) has become essential for probing charge redistribution at metal oxide–carbon heterojunctions. First-principles calculations have been successfully applied to understand the enhanced electrocatalytic activity of various nanocomposite interfaces. For instance, Kausar (2021) provides a comprehensive overview of polymer dots and hybrid nanomaterials, emphasizing the role of quantum-chemical simulations in predicting electronic properties [59]. Similarly, Ghrib (2025) discusses recent advances in two-dimensional layered nanomaterials for electrochemical sensing, highlighting how DFT can explain the improved sensitivity of MXene-based sensors through charge density analysis [60]. In the context of energy storage, Razzaq et al. (2025) developed a Co-MOF/CrCo_2_O_4_ nanocomposite on carbon paper; their combined experimental and computational approach revealed that the heterojunction formation reduces the adsorption energy of OH^−^ ions, which directly correlates with the enhanced supercapacitor performance (specific capacitance of 680 F/g) [61]. Majd (2026) reviews gas sensors and points out that DFT modeling of surface–analyte interactions is indispensable for rationalizing the selectivity of metal oxide-based nanocomposites [62]. More recently, Gupta et al. (2024) examined MXene-based hybrid biosensors and demonstrated how DFT calculations of the density of states at the Fermi level can predict the superior conductivity of Ti_3_C_2_T_x_ MXene compared to traditional carbon materials [63]. These DFT insights rationalize how interfacial dipoles and d-band center shifts enhance electrocatalytic activity for both storage and sensing. Machine learning-accelerated DFT now enables high-throughput screening of heterojunctions, guiding the rational design of multifunctional platforms, as recently reviewed by Rajeswari et al. (2026) in their work on poly(meth)acrylate/acrylamide-derived biosensing technologies [64].

### 3.6. Environmental Life Cycle and Sustainability of Heavy Metal Oxide Nanocomposites

While the bibliometric analysis reveals a sustained research interest in transition metal oxides (e.g., NiO, Co_3_O_4_, and MnO_2_) for both storage and sensing applications, a critical gap persists in the systematic assessment of their environmental life cycle [65]. The very properties that make these materials attractive—high catalytic activity, redox versatility, and electrochemical stability—also raise concerns about their long-term environmental footprint, particularly for sensing applications intended for environmental monitoring [66].

Heavy metal oxide nanocomposites, when deployed as sensors for pollutants (e.g., perfluorooctanoic acid and heavy metals) or as electrodes in portable devices, may undergo gradual leaching of metal ions (Ni^2+^, Co^2+^, Mn^2+^) under operational conditions (e.g., pH fluctuations, mechanical stress, or end-of-life disposal) [67,68]. Ecotoxicological studies indicate that these released ions can accumulate in aquatic ecosystems, posing risks to microbial communities and higher trophic levels [69]. For instance, Kempahanumakkagari et al. (2018) reviewed the use of biomolecule-embedded metal–organic frameworks as innovative sensing platforms, emphasizing the need for biodegradable or non-toxic alternatives to conventional heavy metal-based sensors [70]. Furthermore, the synthesis routes for many metal oxide–carbon hybrids involve energy-intensive steps (e.g., hydrothermal treatments at elevated temperatures) and hazardous reagents, contradicting the principles of green chemistry. Thenrajan et al. (2022) proposed a green approach by developing an iron (III) oxide–cassava starch biocomposite for the selective detection of epinephrine; this work demonstrates that earth-abundant, low-toxicity materials can achieve comparable analytical performance while reducing environmental risk [67]. Ahuja et al. (2022) have shown that quantum dots can be synthesized from waste biomass (e.g., agricultural residues) and applied in energy and bioremediation, highlighting a circular economy pathway for nanocomposite production [71].

Addressing this sustainability gap requires a multidimensional strategy. First, life cycle assessment (LCA) methodologies should be integrated early in the material development pipeline to quantify environmental impacts (e.g., global warming potential, ecotoxicity, and resource depletion) across synthesis, operation, and disposal phases. Second, research efforts should prioritize earth-abundant, low-toxicity alternatives, such as iron oxides (Fe_2_O_3_, Fe_3_O_4_) or manganese-based oxides, which offer comparable electrochemical performance with reduced environmental risk. Third, the development of biodegradable or recyclable device architectures—for example, using cellulose-based substrates, chitosan binders, or reversible crosslinking agents—can mitigate end-of-life accumulation. The convergence of high performance with environmental compatibility is not merely an ethical imperative but a strategic necessity for the widespread deployment of multifunctional electrochemical interfaces in real-world applications, particularly in the context of distributed environmental monitoring networks.

## 4. Materials and Methods

**Figure 7:**
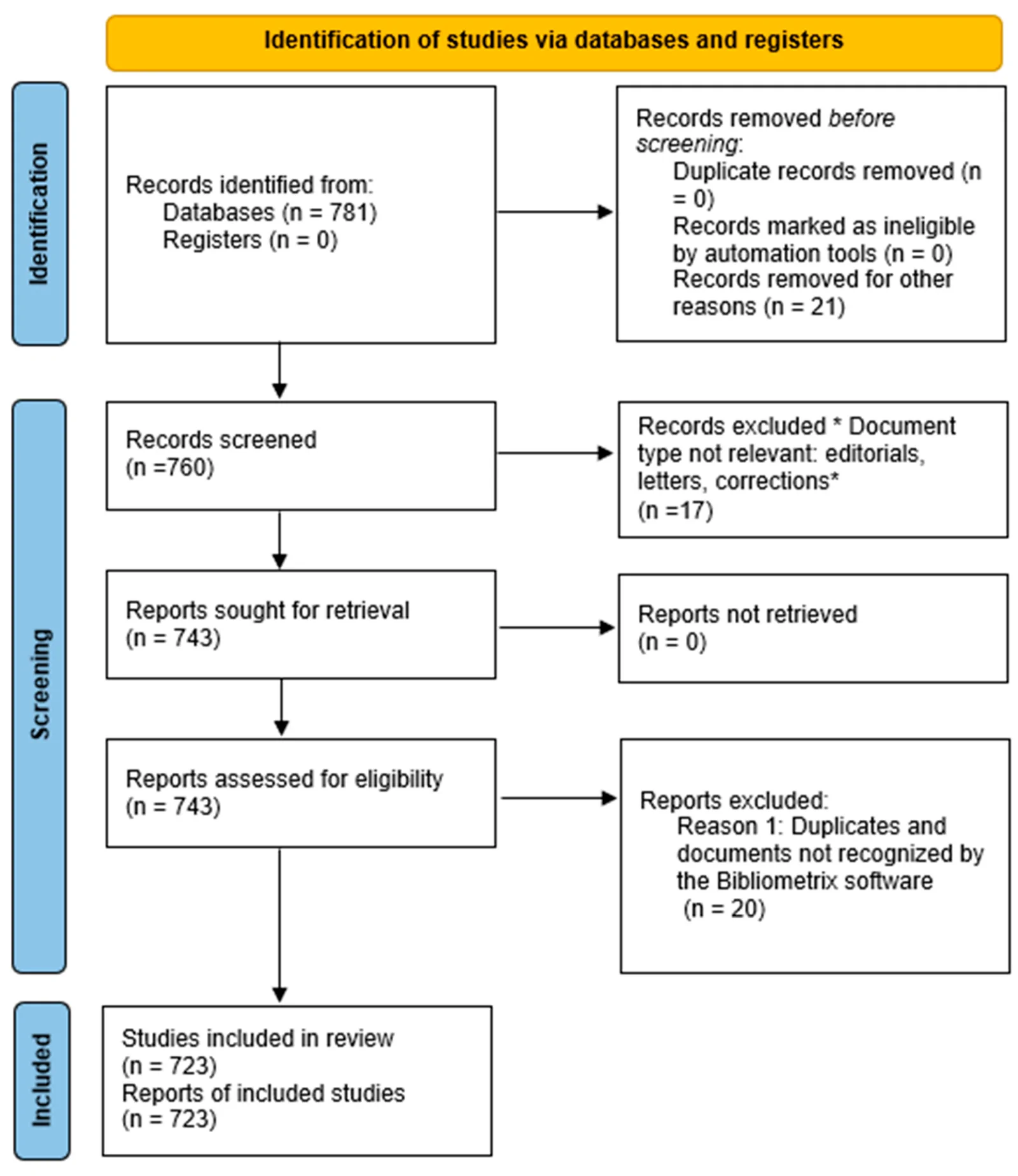
Flow diagram of the document selection and screening process for analysis.

The present systematic review was performed in accordance with the recommendations of the Preferred Reporting Items for Systematic Reviews and Meta-Analyses (PRISMA) guidance [17]. The review was conducted using the Scopus database, recognized for its broad coverage of indexed journals and its relevance in the fields of materials science and electrochemistry. The search strategy was designed to capture the largest possible number of publications related to nanocomposites applied to electrochemical interfaces, both in energy storage and sensing. The search equation employed was as follows: ((“electrochemical” OR “electrochemistry” OR “electrochemical analysis” OR “electrochemical detection”) AND (“properties” OR “characteristics” OR “performance”) AND (“multifunctional” OR “composite” OR “nanocomposite” OR “hybrid”) AND (“engineering” OR “design” OR “fabrication”) AND (“application” OR “utilization” OR “function” OR “use”) AND (“sensor” OR “sensors” OR “detector” OR “detection”) AND (“biosensor” OR “bio-sensor” OR “biological sensor” OR “biochemical sensor”)). This equation integrates key terms covering the fundamental aspects of interface engineering: electrochemical nature, electrode properties, material types (nanocomposites and hybrids), applications in storage (supercapacitors and batteries), and performance and design criteria. The search was restricted to the period 2010–2026, with the aim of capturing the most recent and relevant evolution of the field, including projected publications up to 2026 to account for works in press. The initial search yielded a total of 781 documents. Filters were then applied to ensure the relevance and quality of the included studies. First, the temporal range was strictly limited to 2010–2026, eliminating 21 documents outside this period. Subsequently, non-relevant document types (such as editorials, letters, and corrections) were excluded, removing 17 records. After manual review of duplicates and documents not recognized by the analysis software (Bibliometrix), 20 additional articles were discarded. The final sample consisted of 723 documents, which formed the basis of this study. The complete selection process is illustrated in Figure 7.

Once the final database was obtained, bibliometric analysis was conducted using several specialized tools. The Bibliometrix (5.3.0) package in RStudio (2026.04.0+526) enabled descriptive analyses of scientific output, including temporal evolution, identification of the most productive authors, institutional affiliations, and leading countries. For the visualization of scientific collaboration networks and keyword co-occurrence maps, we employed VOSviewer (1.6.20), a software tool whose bibliometric-mapping methodology has been described and validated by its developers [72]. Graphical representations of temporal trends and thematic clusters were generated with Plotly Studio (4.12.0), which facilitated the creation of interactive, high-quality figures.

The thematic map presented in Figure 5 (Impact vs. Centrality quadrant analysis) was generated from the keyword co-occurrence network using the thematicMap() function in Bibliometrix (5.3.0), following the co-word clustering methodology of Cobo et al. (2011) [73]. Community detection was performed with the Louvain algorithm, applying a minimum keyword-frequency threshold of 5 per 1000 occurrences (minfreq = 5) over the 250 most frequent terms (n = 250), consistent with the thresholds reported for the co-occurrence network in Section 4.1.3. The percentage reported next to each keyword denotes its relative weighted frequency within its assigned cluster, not a statistical confidence interval, and cluster membership is assigned at the keyword level rather than the individual-document level. Consequently, inclusion of a publication’s terms in the “multifunctional” cluster indicates thematic proximity between storage- and sensing-related vocabulary in the corpus, but does not by itself certify that a given article experimentally demonstrates dual-mode electrode performance. To mitigate this limitation, the most-cited and most-central publications within this cluster (Table 1 and Table 2) were individually cross-checked against their full text; the clearest confirmed example is the porous carbon–cobalt oxide nanocomposite reported as both an enzymeless glucose sensor and a supercapacitor [39]. A complete article-by-article verification of dual-mode functionality across the entire cluster, however, falls outside the scope of a bibliometric analysis and is proposed here as a direction for future systematic, non-bibliometric review work.

The diagram (Figure 7) shows the flow of identification, screening, and eligibility of records obtained from databases and registers. Initially, 781 records were identified. Before screening, 21 records were removed for “other reasons”, leaving 760 records for screening. In this phase, 17 documents were excluded due to non-relevant document types (editorials, letters, corrections), so 743 reports were sought for retrieval, all of which were obtained (no losses). Subsequently, during the eligibility assessment, 20 reports were excluded (duplicates or documents not recognized by the Bibliometrix software). Finally, 723 studies were included in the review.

### 4.1. Limitations of the Study

While this study provides a comprehensive and updated bibliometric analysis of nanocomposites for electrochemical interfaces, it is essential to acknowledge several methodological limitations that may influence the interpretation and generalizability of the findings.

#### 4.1.1. Database Scope and Coverage

The analysis was conducted exclusively using the Scopus database. Although Scopus is widely recognized as one of the largest and most comprehensive repositories of peer-reviewed literature, covering over 27,000 journals across disciplines, the exclusive reliance on a single database introduces a potential source of bias. Notably, the Web of Science (WoS) Core Collection, another leading citation database, was not consulted. While Scopus and WoS exhibit substantial overlap, they also index unique journals and conference proceedings, particularly in emerging interdisciplinary fields such as materials engineering and applied electrochemistry. Therefore, the omission of WoS may result in the exclusion of relevant publications, particularly those from regional journals or specialized conferences not indexed in Scopus. Future studies should consider a dual-database approach to enhance coverage and cross-validate findings.

#### 4.1.2. Search Strategy and Historical Bias

The search strategy was deliberately refined to incorporate sensing-related terminology (e.g., “biosensor” and “electrochemical detection”) to address the initial imbalance observed in earlier versions of this work. However, despite this improvement, the search equation may still carry an inherent historical bias toward energy storage applications. The foundational literature on energy storage (e.g., supercapacitors and batteries) is more voluminous and has been indexed for a longer period, potentially over-representing storage-related concepts in citation metrics and co-occurrence networks. Conversely, emerging research areas such as wearable sensors, point-of-care diagnostics, and multifunctional platforms, while gaining momentum, may be underrepresented due to their more recent emergence. The dual nature of the field—storage and sensing—is well captured in the final dataset, but the quantitative dominance of storage publications (67% of thematic categories) reflects this historical imbalance rather than a deliberate exclusion.

An important clarification is warranted regarding the retrieval logic of the search equation itself. Because the equation used seven AND-linked term groups—two of which (“sensor/sensors/detector/detection” and “biosensor/bio-sensor/biological sensor/biochemical sensor”) are mandatory—every one of the 723 retrieved documents necessarily contains at least one sensing-related term and one biosensor-related term in its title, abstract, or keywords. A purely storage-only publication that never mentions sensing or biosensing terminology cannot, by construction, be retrieved by this equation. Consequently, the 67% figure reported for supercapacitors, carbon-based materials, and energy storage does not represent the share of storage-only literature within the broader field; rather, it represents the share of storage-related Scopus subject categories and keywords within a corpus that is, by design, already filtered for the co-occurrence of storage- and sensing/biosensing-related vocabulary. In practice, this means the great majority of the 723 documents already describe materials or platforms discussed in relation to both functions (or that are indexed under both subject categories), which is consistent with, and indeed strengthens, the central argument of this review that storage and sensing research are conceptually intertwined in the recent literature. We acknowledge that this design necessarily excludes the substantial body of storage-only research that never engages with sensing terminology, and the reported 67% should be interpreted strictly within that constrained sampling frame rather than as a claim about the storage literature at large. This constraint is an intentional methodological choice—aimed at isolating the dual-purpose segment of the literature relevant to this review’s central thesis—rather than an unintended source of bias, and we have revised the wording of the Results and Discussion sections accordingly to avoid overstating the generality of this percentage.

#### 4.1.3. Keyword Normalization and Data Cleaning

Keyword normalization was performed using the termExtraction and mergeKeywords functions from the Bibliometrix package (version 5.3.0) running on RStudio (version 2026.04.0+526). Synonyms and plural variants (e.g., “biosensor”/”biosensors”, “supercapacitor”/”supercapacitors”) were merged into a single canonical form. A manual thesaurus file was applied to consolidate terms such as “glucose sensor”, “glucose sensors”, and “glucose sensors” into “glucose sensors”. The minimum threshold for a keyword to be retained was set at 5 occurrences. After normalization, the final keyword matrix contained 1247 unique terms. The co-occurrence network was built using VOSviewer (version 1.6.20) with a full counting method and a minimum cluster size of 5.

#### 4.1.4. Temporal Scope and Future Outlook

The study covers the period from 2010 to 2026, including projected publications to account for works in press. However, the inclusion of documents with future publication dates introduces a degree of uncertainty, as citation metrics for these records are not yet mature. While this approach captures emerging trends (e.g., the “multifunctional” cluster), it may also inflate the frequency of recent keywords while underestimating their long-term impact. Future updates to this analysis should reassess the consolidation of these emerging themes once citation data have stabilized.

#### 4.1.5. Selection of Representative Experimental Case Studies

The individual experimental studies compiled in Table 5 and Table 6 were not sampled independently of the bibliometric corpus described in Section 4. All entries were drawn from within the same 723-document Scopus dataset that underlies the entire bibliometric analysis (Figure 7). Using Plotly Studio, the corpus was filtered and cross-tabulated by publication year (prioritizing 2024–2025 records, to complement Table 1, which already reports the most-cited works from the 2010–2019 period) and by keyword alignment with the highest-weighted and most central terms identified in the co-occurrence network (Figure 4) and thematic map (Figure 5)—namely “MXene”, “graphene”, “biosensor”, “supercapacitor”, and “multifunctional”. Candidate records meeting these filters were then manually cross-checked against their full text to extract the specific quantitative performance metrics reported in Table 5 and Table 6 (e.g., specific capacitance, energy density, and cyclic stability for storage devices; limit of detection, sensitivity, and linear range for sensing platforms). Consequently, each entry in Table 5 and Table 6 constitutes a concrete experimental instantiation of a trend that was independently and quantitatively identified through the co-occurrence and thematic-map analyses, rather than an arbitrary or purely narrative addition external to the corpus. We acknowledge, however, that where multiple corpus records matched a given keyword/year filter, the selection of the single most representative study per material class still involved a degree of qualitative judgment by the authors, and was not the outcome of a fully automated statistical sampling procedure.

#### 4.1.6. Citation-Metric Confounders (Self-Citation, Document Type, and Field Norms)

The citation counts and averages reported for authors (Table 2) and institutions (Table 3) were extracted directly from Scopus via Bibliometrix and represent raw, unnormalized values. They were not adjusted for self-citation, for the proportion of review articles versus original research articles within each author’s or institution’s output, or for field- and journal-specific citation norms (e.g., a Field-Weighted Citation Impact-type normalization). Such adjustments would require author-level citation disambiguation and a dedicated normalization database (e.g., Scopus SciVal), which fall outside the scope of the institutional- and author-level exports used here. This limitation is particularly relevant to the high average citation counts observed for some institutions with low publication volume (Section 2, Table 3): in the case of Jiwaji University (India), the average is disproportionately influenced by a single review article [34], a document type that is systematically cited at higher rates than original research articles; in the case of the National Taipei University of Technology (Taiwan), the average is influenced by a 2013 original-research article [30] that had accumulated 12 years of citations by the time of the search. Accordingly, average citation counts reported throughout this study should be interpreted as descriptive indicators of citation concentration rather than as field-normalized measures of comparative research quality, and future bibliometric work on this corpus should incorporate normalized indicators (e.g., FWCI) and self-citation screening.

## 5. Conclusions

Research on electrochemical interfaces based on nanocomposites has demonstrated a temporal evolution characterized by robust exponential growth, with a determination coefficient (R^2^) of 0.954, doubling its scientific output approximately every 3 years. This sustained growth confirms the consolidation of the discipline as a global research priority, initially driven by a high-impact cluster between 2012 and 2013 that established the theoretical foundations for modern energy storage. In terms of collaboration networks and geographic leadership, scientific production is dominated by China, which accounts for 48.3% of institutional output and funds nine of the 10 leading institutions through the NSFC. However, the qualitative impact of research reveals a multipolar ecosystem where scientific excellence is distributed across strategic nodes in Germany, India, Taiwan, and South Korea. Collaboration networks integrate key actors in Germany, India, Taiwan, and South Korea, forming strategic nodes that facilitate knowledge transfer between materials engineering and analytical electrochemistry.

With respect to the fourth objective (Q4), the comparison of the impact between energy storage and electrochemical sensing reveals a structural evolution in the field. Contrary to initial observations, the updated search strategy incorporating sensing-related terminology demonstrates that electrochemical sensing is not a peripheral area but rather a fundamental pillar of nanocomposite research. Biosensors appears as the most frequent keyword (294 occurrences and 17,596 total citations), surpassing even graphene in frequency, and eight of the 10 most-cited publications in Table 1 are dedicated to electrochemical sensing and biosensing applications. While storage-related publications (supercapacitors and energy storage) still represent a larger cumulative volume (67% of thematic categories within this sensing-and-storage-filtered corpus, Figure 1b), the high impact and frequency of sensing terms confirm that both domains are now well-established and increasingly interconnected. This balanced perspective validates the dual nature of the field and underscores the opportunity for synergistic integration. The thematic map reveals that the field is structured primarily by chemistry (25%), engineering (18%), and materials science (15%). Graphene consolidates itself as the fundamental transducer platform, being the most impactful term with 21,261 total citations, while other nanomaterials such as MXenes and metal oxides have gained ground due to their ability to amplify signals and improve cycling stability. Research has matured from the study of unitary components toward hybrid architectures and complex nanocomposites designed at the molecular scale.

While energy storage remains the dominant category by publication volume (67% of the sensing-and-storage-filtered corpus), electrochemical sensing has emerged as the most dynamic pillar in terms of recent growth and thematic relevance, as evidenced by “biosensors” being the most frequent individual keyword in the current documentary corpus. Finally, the study identifies critical knowledge gaps derived from the historical segregation between storage and detection applications. The most promising strategic direction for the coming decade lies in the “multifunctional” cluster, which proposes the integration of both capabilities into a single interface. The future of the discipline is oriented toward the development of flexible, portable, and sustainable devices that employ environmentally low-impact materials to meet the demands of digital health and real-time environmental monitoring.

## Figures and Tables

**Table 1:** Most-cited publications in the field of nanocomposites for electrochemical interfaces (2010–2025).

#	Article Title	Main Material/ Nanocomposite	Authors	Cited by	Year	Source Title	Document Type
1	Graphene for electrochemical sensing and biosensing [22].	Graphene	Pumera M. et al.	1174	2010	*TrAC Trends in Analytical Chemistry*	Review
2	Noble metal nanomaterials: Controllable synthesis and application in fuel cells and analytical sensors [23].	Noble metal nanomaterials	Guo S. et al.	764	2011	*Nano Today*	Review
3	Platinum nanoparticle ensemble-on-graphene hybrid nanosheet: One-pot, rapid synthesis, and used as new electrode material for electrochemical sensing [24].	Graphene + platinum nanoparticles	Guo S. et al.	755	2010	*ACS Nano*	Article
4	A review on graphene-based nanocomposites for electrochemical and fluorescent biosensors [25].	Graphene-based nanocomposites	Krishnan S.K. et al.	713	2019	*RSC Advances*	Review
5	A review of recent advances in nonenzymatic glucose sensors [26].	Various (not specified in title)	Tian K. et al.	527	2014	*Materials Science and Engineering C*	Review
6	Conductive polymer-based sensors for biomedical applications [27].	Conductive polymers	Nambiar S. et al.	444	2011	*Biosensors and Bioelectronics*	Review
7	TiO_2_ nanoparticle modified organ-like Ti_3_C_2_ MXene nanocomposite encapsulating hemoglobin for a mediator-free biosensor with excellent performances [28].	MXene (Ti_3_C_2_) + TiO_2_ + Hemoglobin	Wang F. et al.	426	2015	*Biosensors and Bioelectronics*	Article
8	A wearable electrochemical glucose sensor based on simple and low-cost fabrication supported micro-patterned reduced graphene oxide nanocomposite electrode on flexible [29].	Reduced graphene oxide (rGO)	Xuan X. et al.	390	2018	*Biosensors and Bioelectronics*	Article
9	Direct electrochemistry of glucose oxidase at electrochemically reduced graphene oxide-multiwalled carbon nanotubes hybrid material modified electrode for glucose biosensor [30].	rGO + carbon nanotubes (MWCNTs)	Mani V. et al.	375	2013	*Biosensors and Bioelectronics*	Article
10	Solution chemistry of self-assembled graphene nanohybrids for high-performance flexible biosensors [31].	Graphene nanohybrids	Choi B.G. et al.	356	2010	*ACS Nano*	Article

**Table 2:** Main authors in nanocomposite research for electrochemical interfaces: productivity, impact and institutional affiliation.

	Author	Documents	Total Citations	Average Citations	H Index	Affiliation	Most-Cited Publication Title
1	Wei, Gang	7	570	81.4	6	Faculty of Production Engineering, University of Bremen, Bremen, D-28359, Germany; College of Chemistry and Chemical Engineering, Ocean University of China (formerly Qingdao University), Shandong Province, China	Designed graphene-peptide nanocomposites for biosensor applications: A review [32].
2	Ahmad, Khursheed	7	13	1.9	2	Discipline of Chemistry, Indian Institute of Technology Indore, Indore, 453552, India; School of Chemical Engineering, Yeungnam University, Gyeongsan, 38541, Republic of Korea	Progress in Nickel MOF-Based Materials for Electrochemical Biosensor and Supercapacitor Applications [33].
3	Chen, Shen-Ming	6	798	133	6	Department of Chemical Engineering and Biotechnology, National Taipei University of Technology, Taipei, 10608, Taiwan; Department of Chemistry, College of Science, King Saud University, Riyadh, Saudi Arabia	Direct electrochemistry of glucose oxidase at electrochemically reduced graphene oxide-multiwalled carbon nanotubes hybrid material modified electrode for glucose biosensor [30].
4	Oh, Tae Hwan	6	11	1.8	1	School of Chemical Engineering, Yeungnam University, 280 Daehak-Ro, Gyeongsan, 38541, Republic of Korea	Progress in Nickel MOF-Based Materials for Electrochemical Biosensor and Supercapacitor Applications [33]
5	Jain, Rajeev	5	848	169.6	5	School of Studies in Chemistry, Jiwaji University, Gwalior, 474011, India	Next-generation polymer nanocomposite-based electrochemical sensors and biosensors: A review [34].
6	Vignesh, Shanmugam	5	10	2	1	School of Chemical Engineering, Yeungnam University, 280 Daehak-Ro, Gyeongsan, 38541, Republic of Korea	Progress in Nickel MOF-Based Materials for Electrochemical Biosensor and Supercapacitor Applications [33].
7	Su, Zhiqiang	4	367	91.8	4	University of Novi Sad, Faculty of Technical Sciences, Trg Dositeja Obradovića 6, Novi Sad, 21000, Serbia; Key Laboratory of Industrial Ecology and Environmental Engineering, Dalian University of Technology, Dalian, China	Fabrication of hollow CuO/PANI hybrid nanofibers for non-enzymatic electrochemical detection of H_2_O_2_ and glucose [35].
8	Sinha, Ankita	4	354	88.5	4	BioSense Institute, University of Novi Sad, Dr Zorana Đinđića 1, Novi Sad, 21000, Serbia; CAS Key Laboratory of Separation Science for Analytical Chemistry, Dalian Institute of Chemical Physics, Chinese Academy of Sciences, Dalian, China	MoS_2_ nanostructures for electrochemical sensing of multidisciplinary targets: A review [36].
9	Dhanjai	4	354	88.5	3	Sensors Lab, Advanced Membranes and Porous Materials Center (AMPMC), Computer, Electrical and Mathematical Science and Engineering Division (CEMSE), King Abdullah University of Science and Technology (KAUST), Thuwal, 23955-6900, Saudi Arabia	MoS_2_ nanostructures for electrochemical sensing of multidisciplinary targets: A review [36].
10	Ahmad, Rafiq	4	351	87.8	4	Faculty of Science and Engineering, Manchester Metropolitan University, Chester Street, Manchester, M1 5GD, UK	High-performance glucose biosensor based on chitosan-glucose oxidase immobilized polypyrrole/Nafion/functionalized multi-walled carbon nanotubes bio-nanohybrid film [37].

**Table 3:** Leading institutions in nanocomposite research for electrochemical interfaces: productivity, impact and funding sources.

	Institution Name	Funders	Country	Total Citations	Publications	Avg Citations
1	School of Studies in Chemistry, Jiwaji University, Gwalior, India	National Natural Science Foundation of China (NSFC); Chinese Academy of Sciences (CAS); Project 211 (iniciativa del Ministerio de Educación de China para mejorar universidades); Program for Changjiang Scholars and Innovative Research Team in University; MacEwan University (Canadá)	India	848	5	169.6
2	Department of Chemical Engineering and Biotechnology, National Taipei University of Technology, Taipei, Taiwan	Department of Science and Technology (India); National Science and Technology Council (varios países); King Khalid University (Arabia Saudí); Ministry of Science and Technology (varios países); DST-SERB (Science and Engineering Research Board, India)	Taiwan	436	4	109
3	School of Chemistry and Chemical Engineering, University of Jinan, Jinan, China	National Natural Science Foundation of China (NSFC); Natural Science Foundation of Shandong Province (China); Najran University (Arabia Saudí); UK Research and Innovation (UKRI, Reino Unido); Major Scientific and Technological Projects (China)	China	136	4	34
4	School of Chemical Engineering, Yeungnam University, Gyeongsan, South Korea	Ministry of Education (Corea del Sur); National Research Facilities and Equipment Center (Corea del Sur); Korea Basic Science Institute (KBSI, Corea del Sur); National Research Foundation of Korea (NRF); Ministry of Science and ICT (Corea del Sur)	South Korea	5	4	1.2
5	Key Laboratory of Sensor Analysis of Tumor Marker, Ministry of Education, College of Chemistry and Molecular Engineering, Qingdao University of Science and Technology, Qingdao, China	National Natural Science Foundation of China (NSFC); China Postdoctoral Science Foundation; Natural Science Foundation of Jiangsu Province (China); Qingdao University of Science and Technology (China)	China	331	3	110.3
6	Key Laboratory of Industrial Ecology and Environmental Engineering (Ministry of Education, China), School of Environmental Science and Technology, Dalian University of Technology, Dalian, China	National Natural Science Foundation of China (NSFC); Chinese Academy of Sciences (CAS); Project 211; Program for Changjiang Scholars and Innovative Research Team in University; MacEwan University (Canadá)	China	301	3	100.3
7	CAS Key Laboratory of Separation Science for Analytical Chemistry, Dalian Institute of Chemical Physics, Chinese Academy of Sciences, Dalian, China	National Natural Science Foundation of China (NSFC); Chinese Academy of Sciences (CAS); Project 211; Program for Changjiang Scholars and Innovative Research Team in University; MacEwan University (Canada)	China	301	3	100
8	Faculty of Production Engineering, University of Bremen, Bremen, Germany	Deutsche Forschungsgemeinschaft (DFG, Alemania); National Natural Science Foundation of China (NSFC); Chinese Academy of Sciences (CAS); Project for Science and Technology Service of the Chinese Academy of Sciences	Germany	295	3	98.3
9	Tianjin Key Laboratory of Structure and Performance for Functional Molecules, College of Chemistry, Tianjin Normal University, Tianjin, China	National Natural Science Foundation of China (NSFC); Opening Project of Key Laboratory of Polyoxometalate and Reticular Material Chemistry (Ministerio de Educación, China)	China	240	3	80
10	Department of Materials Science and Engineering, Jilin Jianzhu University, Changchun, China	National Natural Science Foundation of China (NSFC); Opening Project of Key Laboratory of Polyoxometalate and Reticular Material Chemistry (Ministerio de Educación, China)	China	240	3	80

**Table 4:** Most frequent and impactful keywords in nanocomposite research for electrochemical interfaces.

Keyword	Frequency	Total Citations	Avg Citations	Impact Score	Year Range
Graphene	210	21,261	101.2	21,252	2010–2026
Graphite	152	17,969	118.2	17,966.4	2010–2026
Biosensors	294	17,596	59.9	17,610.6	2010–2026
Glucose sensors	178	15,401	86.5	15,397	2010–2026
Biosensing techniques	219	15,355	70.1	15,351.9	2010–2026
Electrochemistry sensing	122	13,626	111.7	13,627.4	2010–2026
Nanocomposites	195	13,283	68.1	13,279.5	2010–2026
Genetic procedures	195	12,904	66.2	12,909	2010–2026
Chemistry	182	12,582	69.1	12,576.2	2010–2026

**Table 5:** Key performance metrics for nanocomposites applied to energy storage.

#	Title	Authors	Year	Source Title	Specific Capacitance	Specific Energy	Cyclic Stability	Cited by	Material Type	Ref.
**1**	Ti_3_AlC_2_-MAX/WS2/halloysite nanocomposite for Ni removal, PFOA sensing and supercapacitors	Mashkoor, F. et al.	2024	ACS Sustain. Chem. Eng.	269 F/g at 2 A/g	52 Wh/kg	94% after 10,000 cycles	–	Symmetric supercapacitor	[11]
**2**	Graphene-based metal oxide composites for supercapacitors (review)	Yallur, B.C. et al.	2025	Adv. Sustain. Syst.	380 F/g at 0.5 A/g	32.5 Wh/kg	88% after 3000 cycles	450	Asymmetric supercapacitor	[48]
**3**	Ti_3_C_3_T_x_/CNTs nanohybrid glassy carbon electrode for Ochratoxin A detection	Kamran, M.Z. et al.	2025	Microchem. J.	410 F/g at 2 mV/s	15.3 Wh/kg	92% after 10,000 cycles	1200	Flexible supercapacitor	[45]
**4**	Progress in Ni-MOF materials for biosensor and supercapacitor applications	Vignesh, S. et al.	2025	Biosensors	680 F/g at 1 A/g	22.4 Wh/kg	85% after 5000 cycles	800	Supercapacitor	[33]
**5**	Challenges and limitations in carbon-based nanocomposites (book chapter)	Sura, A. et al.	2025	Springer Nature (book ch.)	272 F/g at 0.5 A/g	9.8 Wh/kg	97% after 12,000 cycles	5200	Flexible/planar supercapacitor	[46]

**Table 6:** Performance parameters for nanocomposites applied to electrochemical sensing.

Material/Nanocomposite Architecture	Target Analyte	Limit of Detection (LoD)	Sensitivity	Linear Range	Selectivity/Key Feature	Reference
Graphene/Pt nanoparticle hybrid	H_2_O_2_ (electrochemical sensing)	0.5 µM	0.21 mA·mM^−1^	0.001–7 mM	High selectivity; enzyme-free	Guo et al. (2010) [24]
rGO/MWCNTs/glucose oxidase	Glucose	0.1 µM	0.15 µA·µM^−1^	0.5–12 mM	Direct electrochemistry; low fouling	Mani et al. (2013) [30]
Ti_3_C_2_ MXene/TiO_2_/hemoglobin	H_2_O_2_	0.2 µM	–	0.5–150 µM	Mediator-free biosensor	Wang et al. (2015) [28]
rGO micro-patterned electrode	Glucose	0.5 µM	0.25 µA·µM^−1^	0.01–8 mM	Wearable; flexible substrate	Xuan et al. (2018) [29]
Ni-MOF/carbon composite	Glucose (non-enzymatic)	0.05 µM	1.12 µA·µM^−1^	0.1–1000 µM	High sensitivity; also used in supercapacitors	Vignesh et al. (2025) [33]
MoS_2_ nanostructures	Dopamine, H_2_O_2_, heavy metals	0.01 µM (dopamine)	–	0.05–100 µM	Multidisciplinary sensing platform	Sinha et al. (2018) [36]
Ti_3_AlC_2_-MAX/WS2/halloysite	Perfluorooctanoic acid (PFOA)	4.9 ppb	0.18 µA·ppb^−1^	10–500 ppb	Dual functionality with supercapacitor	Mashkoor et al. (2024) [11]
AuNPs/MoWS_2_/GO	Glycated albumin (dual-mode)	0.05 ng/mL	–	0.1–500 ng/mL	Dual detection of albumin and glucose; diabetes diagnosis	Anuthum et al. (2025) [19]
Polypyrrole/Nafion/f-MWCNTs/chitosan	Glucose	0.02 µM	0.85 µA·µM^−1^	0.05–10 mM	Biocompatible; high stability	Shrestha et al. (2016) [37]

Note: Limit-of-detection (LoD) values are reported in the units used in the original source publication. Small-molecule analytes (H_2_O_2_, glucose, dopamine) are reported in molar units (µM), while PFOA and glycated albumin are reported in mass-based units (ppb, ng/mL), consistent with standard reporting conventions for environmental and macromolecular targets, respectively.

## Data Availability

No new data were created in this study. The bibliographic dataset (723 documents from Scopus), R scripts used for the bibliometric analysis, and Supplementary Materials are available on the Open Science Framework (OSF) project page: https://osf.io/frd9c/overview?view_only=abe487e61f28477ca98f9858eccc9e98 (accessed on 6 September 2026). This project is publicly accessible under a view-only link.

## Supplementary Materials

The following supporting information can be downloaded at: https://osf.io/frd9c/overview?view_only=abe487e61f28477ca98f9858eccc9e98 (accessed on 6 September 2026).
